# The onset of faba bean farming in the Southern Levant

**DOI:** 10.1038/srep14370

**Published:** 2015-10-13

**Authors:** Valentina Caracuta, Omry Barzilai, Hamudi Khalaily, Ianir Milevski, Yitzhak Paz, Jacob Vardi, Lior Regev, Elisabetta Boaretto

**Affiliations:** 1 Max Planck-Weizmann Center for Integrative Archaeology and Anthropology, 76100, Rehovot, Israel; 2 D-REAMS Radiocarbon Laboratory, Weizmann Institute of Science, 76100, Rehovot, Israel; 3 Israel Antiquities Authority, POB 586, 91400 Jerusalem, Israel

## Abstract

Even though the faba bean (*Vicia faba* L.) is among the most ubiquitously cultivated crops, very little is known about its origins. Here, we report discoveries of charred faba beans from three adjacent Neolithic sites in the lower Galilee region, in the southern Levant, that offer new insights into the early history of this species. Biometric measurements, radiocarbon dating and stable carbon isotope analyses of the archaeological remains, supported by experiments on modern material, date the earliest farming of this crop to ~10,200 cal BP. The large quantity of faba beans found in these adjacent sites indicates intensive production of faba beans in the region that can only have been achieved by planting non-dormant seeds. Selection of mutant-non-dormant stock suggests that the domestication of the crop occurred as early as the 11^th^ millennium cal BP. Plant domestication| *Vicia faba* L.| Pre-Pottery Neolithic B| radiocarbon dating| Δ^13^C analysis.

The shift from foraging to food production, which in many cases marked the transition from a hunter-gatherer to a sedentary lifestyle, brought about substantial changes in the history of humankind and its relation to the ecosystem^1^. The process of plant domestication, based on the selection of phenotypes with characteristics that are more suitable for agriculture, systematically reduced the range of ecotypes available for selection and improvement and led to the loss of ‘wild-type’ traits that ensure the survival of species in their natural habitat. Unraveling the early history of the faba bean would highlight a turning point in human civilization, by documenting the transition from food collection to food production, and could provide new insights that could help improve the crop for the future. We use stable carbon isotope analysis, radiocarbon dating and archaebotany to deepen our understanding of the nature of the selection process that accompanies domestication of the faba bean.

The faba bean is a major crop in many countries, including China, Ethiopia and Egypt, and it is widely grown for human consumption throughout the Mediterranean region and in parts of Latin America^2^. Worldwide it is the third most important feed grain legume after soybean (*Glycine max*) and pea (*Pisum sativum*) and the faba bean is the most efficient N-fixing legume used to reduce the emission of the N_2_O greenhouse gas^3^. Despite its importance, little is known about its wild progenitor and the area of its origin. Cubero^4^ distinguished between four varieties within the domesticated species of faba bean (*major*, *equina*, *minor* and *paucijuga*), and all are interfertile. He suggested the Near East as the core area where *Vicia faba* L. originated, and advocated that *V. faba* var. *major* and *equine* derived from the var. *minor*. Studies of the genetic profile support Cubero’s hypothesis^5^.

To date, the most abundant findings of ancient faba bean (*V. faba* L.) are from the Pre-Pottery Neolithic B (PPNB) site of Yiftah’el, in the Southern Levant, and they were radiocarbon dated to the 10^th^ millennium cal. BP^6^. While faba bean are plentiful at Yiftah’el, very few faba beans were recovered in other PPN sites in the Levant (Fig. 1). This situation changed when relatively large amounts of faba beans were found in Early PPNB contexts at the site of Ahihud (*n* = 6205), and the Middle PPNB sites of Nahal Zippori 3 (*n* = 132) and Yiftah’el (*n* = 1069) (Fig. 2) (Supplementary information)^7^,^8^,^9^. These discoveries were the motivation for the present study on the origins of farming the faba bean.

The large numbers of specimens found and the geographical proximity of the 3 sites of Ahihud, Nahal Zippori 3 and Yiftah’el (Fig. 1b), that therefore share the same environmental conditions, offered a unique opportunity to study the beginning of cultivation of this legume and its process of domestication in the southern Levant. To date the study of domestication of legumes has been based on visible changes of the plant, namely an increase of seed size and a reduction of the natural dispersal mechanism of the seeds (i.e. dehiscent pods). Since pods are rarely found in archaeological contexts, seed size has been considered the best trait to identify domesticated legumes^10^. Here we test the assumption that size is the main change that occurs at the early stage of domestication by measuring biometrical parameters and biochemical properties of the archaeological seeds. Experiments were carried out on modern faba bean to estimate the variability of size due to charring. We used standard size analysis to measure the biometric traits of charred faba beans from the five contexts in Ahihud, Nahal Zippori 3 and Yiftah’el. High-precision radiocarbon dating was used to assess the absolute chronology of seeds from each context, while stable carbon isotopes (Δ^13^C) were measured to infer information on the water input of the ancient seeds during their life cycle^11^,^12^,^13^ and test the correlation between water status and size.

## Results

### Charring experiments on modern material

**Figure 3:**
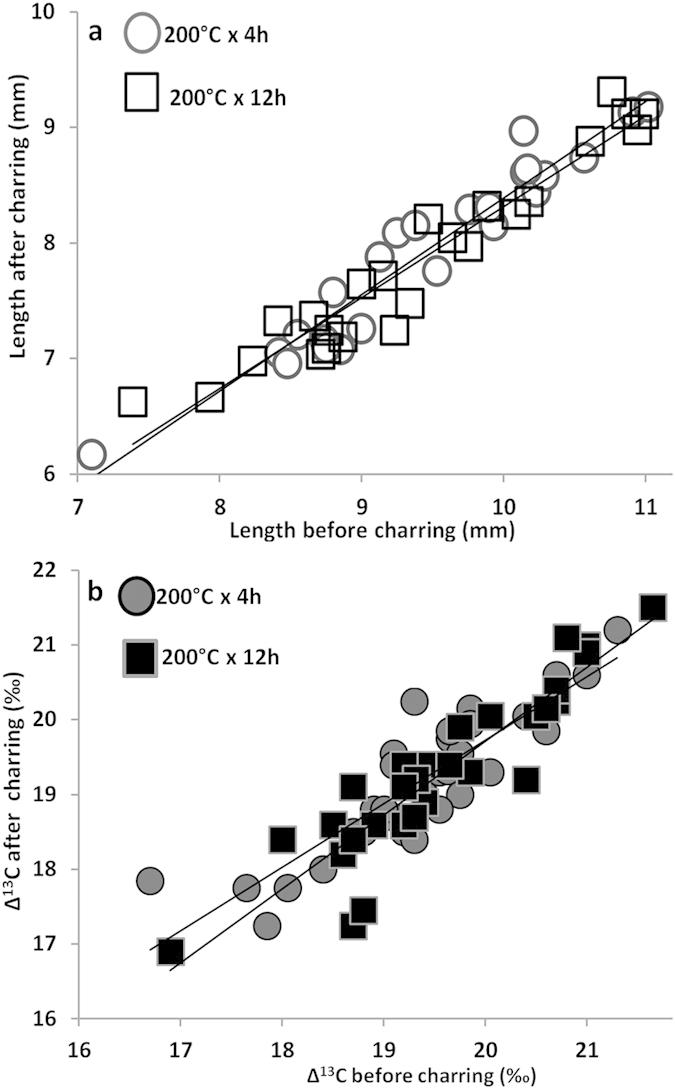
Effect of charring on the modern faba bean. (**a**) Correlation between the length before and after charring at 200 °C × 4 h (24 seeds) and at 200 °C × 12 h (24 seeds). (**b**) Correlation between the Δ^13^C before and after charring at 200 °C × 4 h (32 seeds) and at 200°C x 12h.

The first question we address is the effect of charring on faba beans. The experiment was carried out on 120 modern seeds charred at various temperatures and for different times (Supplementary Table S1). We discovered that modern seeds explode above 200 °C, and therefore the ancient seeds must have been charred at ~200 °C or below. At 250 °C 33% of seeds explode, and at 300 °C all of them explode. The length decreased by ~17%, for both short and long periods of charring (R^2^: 0,93 for 4 h and R^2^: 0,93 for 12 h) (Fig. 3a). Breadth and thickness were also measured but they were found subjected to substantial changes when burned longer than 4 h so they were considered less relevant than length for biometric analysis.

Thus charring results in a homogeneous reduction of length, while breadth and thickness change in an unpredictable way. The charring experiment also showed that the stable carbon isotopic composition (Δ^13^C) is retained in seeds after charring at 200 °C × 4 h or at 200 °C × 12 h (R^2^: 0,77; R^2^: 0,84;) (Fig. 3b) (Supplementary Table S2). We thus conclude that length and stable carbon isotopic composition can provide reliable information on the archaeological samples.

### Size analysis of archaeological material and radiocarbon dating

**Figure 4:**
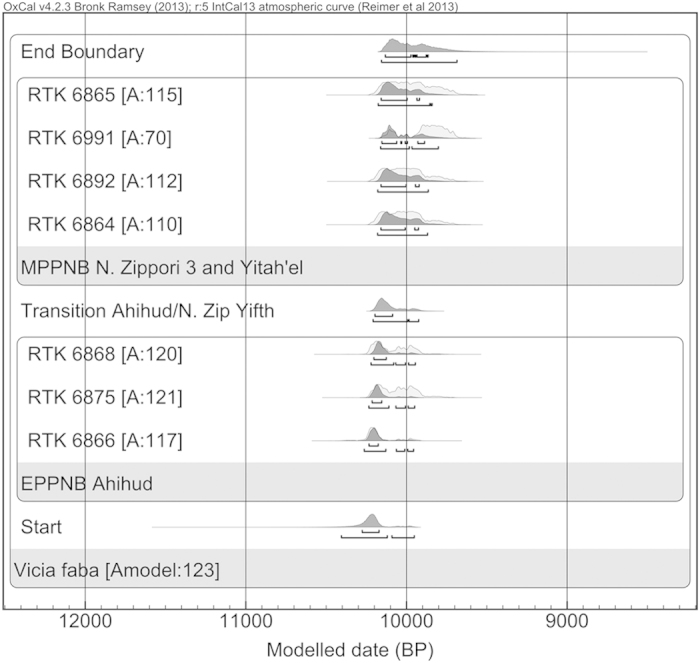
The Bayesian model based on the sequence of the Early PPNB and Middle PPNB dates. The RTK is the laboratory identification label, while the number that follows the label represents the identification number. The samples from Ahihud are RTK 6868, 6875, 6866; Nahal Zippori 3 RTK 6864,6865) and Yiftah’el RTK 6991, 6892. All the dates were measured on *Vicia faba* L. seeds. Dark gray curves are the modeled calibrated 14C range distribution. Light gray curves are the 14C calibrated range distribution.^54^,^55^

**Figure 5:**
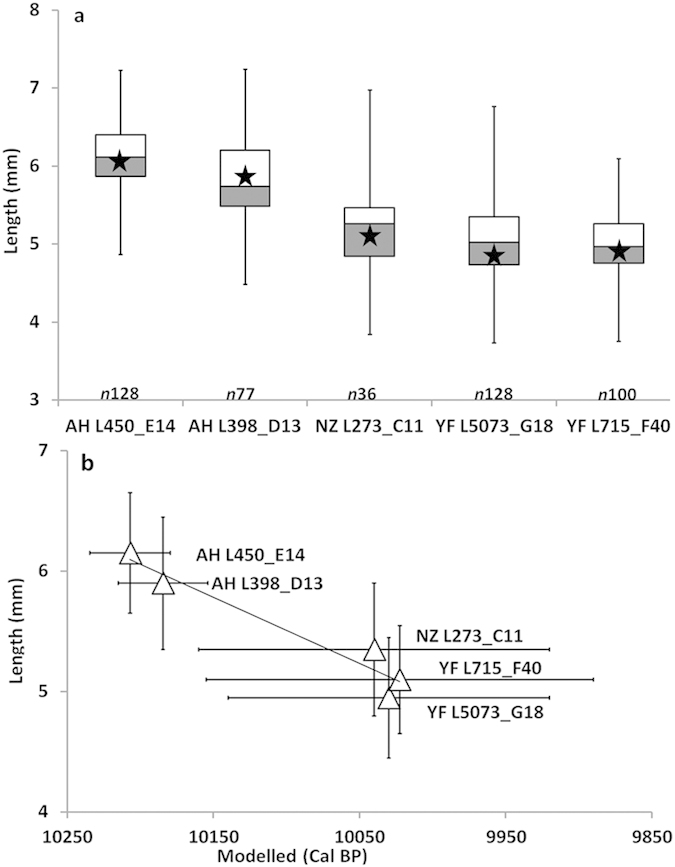
The length of faba bean over the time. (**a**) (*n*) is the number of beans measured, the bottom whisker represents the minimum, the bottom line of the lighter box represents the first quartile (25th percentile), the top line of the darker box represents the third quartile (75th percentile) and in between them is the median line (50th percentile). The top whisker represents the maximum. The star represents the average length of each group of seeds. (**b**) Each triangle represents the average length of the faba beans from the five investigated contexts (AH = Ahihud, NZ = Nahal Zippori, YF = Yiftah’el). The plot shows the decrease of length as a function of time (R^2^: 0,98).

The sizes of 469 seeds collected from two storage pits in Ahihud (Locus 450, SquareE14 and Locus 398, Square D13), a plaster floor in Nahal Zippori 3 (Locus 273, Square C11) and two different contexts in Yiftah’el (Locus 715, Square F40 and Locus 5073, Square G18) were measured (Supplementary Table S3) The samples from Ahihud (Early PPNB) were significantly longer (~20%) than those from Nahal Zippori 3 and Yiftah’el (Middle PPNB) (p < 0,001) (Supplementary Table S4). The relative chronology, based on the associated lithics found in the sites, attributes Ahihud to the Early PPNB, while the other two sites are from the Middle PPNB^7^,^9^. Radiocarbon dates of faba beans from the three sites are consistent with this relative chronology. A simple Bayesian sequence of ^14^C dates shows that Ahihud dates to 10,235–10,125 cal BP and falls at the end of the Early PPNB (Fig. 4). These dates are older than the ones from Nahal Zippori 3 and Yiftah’el, that both dated to 10,160–9,890 cal BP, which fall in the beginning of the Middle PPNB^8^ (Supplementary Table S5). When the sizes of these samples are plotted on an absolute chronological scale, it is clear that the longer faba beans from Ahihud are significantly older than the shorter ones from the other sites (R^2^: 0,98) (Fig. 5a,b).

### *Δ* ^13^C analyses of archaeological faba beans

**Figure 6:**
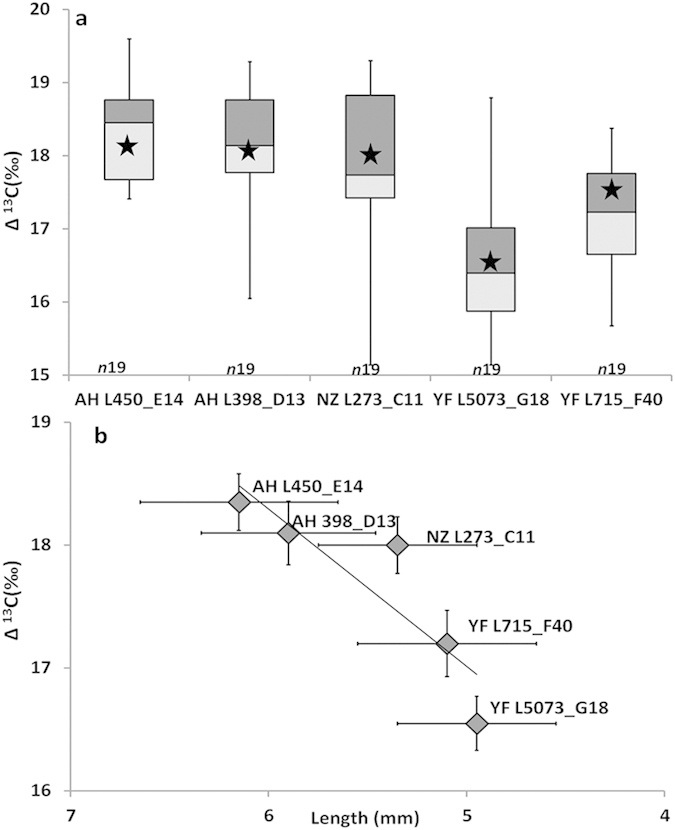
The Δ 13C of faba bean over the time. (**a**) *n*) is the number of seed measured, the bottom whisker represents the minimum, the bottom line of the lighter box represent the first quartile (25th percentile), the top line of the darker box represent the third quartile (75th percentile) in between them is the median line (50th percentile). The top whisker represents the maximum. The star represents the average length of each group of seeds. (**b**) Each square represents the average Δ^13^C of the faba beans from the five investigated contexts (AH = Ahihud, NZ = Nahal Zippori, YF = Yiftah’el). The plot shows the Δ13C (‰) as function of length (R^2^: 0,72).

In order to understand the influence of edaphic conditions on the bean size, we analyzed the stable carbon isotope ratio (Δ^13^C) of 95 archaeological faba beans. The Δ^13^C was previously shown to be a reliable parameter to ascertain the amount of water received by the faba bean during its growth^14^. The samples from Ahihud (Early PPNB) had higher values of Δ^13^C when compared with those of Nahal Zippori 3 and Yiftah’el (Middle PPNB) (p < 0,001). A positive correlation was found between the average length and the average Δ^13^C of each group (R^2^: 0,72) and this shows that the size mainly depends on water availability at the site of growth (Supplementary Table S6) (Fig. 6a,b). This result is in agreement with the hypothesis that the water received during the period of growth accounts for the size of the beans.

## Discussion

### Domestication of legumes

The distinction between cultivation and domestication of the most common edible plants is controversial. Some favor the opinion that a regime of tilling, sowing and reaping (cultivation) acts as a selective force on wild plants, selecting for mutations adapted to the new environmental conditions^15^,^16^. Others claim that mutations that are favorable for agriculture (domestication) must have been selected before the crop could be successfully cultivated^17^,^18^. The mutations typically include reduced seed dormancy, the loss of dispersal mechanisms (indehiscent pods)^18^, reduced seed coat thickness^19^, and increased seed size^10^. The first two mutations do not leave visible traces on the legumes, but wherever large stocks of legumes are found, plants with domestic-traits (non dormant/non dehiscent) must have been used^16^. Furthermore, experiments conducted on wild modern pea, chickpea and lentil prove that neither harvesting of wild stands or cultivation of wild legumes results in profitable yields^17^,^18^,^20^. The thickness of seed coat should be thinner and smoother in domesticated stocks to facilitate water penetration and germination^19^. The validity of this mutation as a trait of domestication remains controversial, because no great differences were found between the domesticated species of lentil, bitter vetch, grass pea and their wild relatives^21^,^22^. Increase in seed size is considered to be one of the major domestication-traits in crops, but analyses conducted on archaeological legumes such as pea, lentil and cowpea show that the increase in seed size does not occur at the early stage of domestication but rather later as a result of crop improvements^23^,^24^.

### Cultivation versus domestication of the faba bean

Scholars that invoke a protracted process of cultivation as an unconscious cause of domestication stress three mechanisms that should increase the seed yield: the seedling depth^15^, ploughing^25^ and selection of larger seeds for seeding^26^. It holds that seeds buried deeper by human planting develop larger reserves because they emerge from a greater depth^27^. In the case of the faba bean, greater burial depth does not increase seed size, while maximum yield is obtained when the seeds are buried no deeper than 8 cm. Faba bean, as well as chickpea and lentil, has an hypogeal germination, meaning that its cotyledon remains where the seed is sown while only the shoot emerges from the soil surface; as a consequence, the seed must be buried close to the surface to sprout above the ground^28^,^29^,^30^.

A negative correlation was also found between tillage and yields^31^. Agronomists agree that tillage does not help to maximize the faba bean yield once the crop is farmed under dry climatic conditions. The relatively shallow root system of faba bean relies on the water accumulated within the first 30–40 cm from the soil surface; therefore soils that have a more stable structure prevent water from percolating to greater soil depths. Another advantage of compacted, non-tilled soils is that residues of crops remain on the surface and this prevent excessive water evaporation, which is a major constraint for plants growing in dry and semi-dry environments^32^. The selection of larger seeds for seedling is commonly considered a profitable way to obtain plants that have bigger seeds and produce higher yields^33^. Thus a relation between seed yield and seed size anticipates that seed size should have a positive influence on seed yield. Nonetheless such a correlation has not been recorded for the faba bean. On average, medium and small size grains of faba bean produce maximum seed yield compared to larger seeds^29^,^34^. The explanation for such a contradictory result is that plants originating from smaller seeds produce a greater number of pods, which are, on average, longer that those produced by plants originating from large seeds. Plants originating from small faba beans sprout, flower, form pods and mature faster, leading to the highest harvest index values. Small-sized grains produce good yield over a range of seasonal conditions, while large seeds are more sensitive to adverse seasonal conditions such as drought and low temperature^29^.

The agronomic studies show that common practices associated to cultivation do not lead to higher yields. Therefore it is unlikely that cultivation acted as a selective force in the process of domestication of the crop. Other mechanisms must have been adopted in order to transform the faba bean into a crop. Seed dormancy (and pods dehiscence) is a typical trait of the wild progenitors of the major legumes (i.e. *Lens culinaris* ssp. *orientalis*, *Pisum humile*, *Cicer reticulatum*, *Vigna radiata* subsp. *sublobata* and *Phaseoulus vulgaris*), so it is safe to assume that unknown progenitor of faba bean would also have some mechanism to delay the germination.

The problem of dormancy in wild legumes can be overcome by soaking the seeds in water (or abrade the seed coat, increase the temperature etc) to encourage the germination of the legumes; thus, repeated use of free-geminating seeds would saturate the natural gene bank with non-dormant seeds in a relatively short time^15^. Another option includes the inadvertent selection of domestication traits by means of cultivation of wild stands of plants; this could also have resulted in the widespread introduction of non-dormant seeds in the natural gene pool, but only after 5–6 cycles of (unprofitable) cultivation^18^.

Regardless of whether the selection of the original seeds had been ‘conscious’, through the selection of non dormant seeds straight from the wild stands, or ‘unconscious’, by inducing phenotopic changes in the local gene pool, the loss of seed-dormancy is the only circumstances that enables the production of legumes in large quantities.

The presence of a large quantity of seeds in Ahihud and Yiftah’el reinforce the idea that non-dormant (and non-dehiscent) stocks were used to ensure reliable harvesting, resulting in the build-up of characteristic domestication traits. The size of the archaeological faba bean cannot be used as a domestication trait, because the Δ^13^C shows that size depends on the amount of water received by the plant. We therefore rely on other parameters to assess domestication. The presence of storage facilities found in Ahihud and Yiftah’el , as well as the presence of other crops in great quantities, such as the 7,2 kg of lentils found in Yiftah’el^35^, are consistent with the notion that the surplus was kept for seeding, to ensure the continuity of legume production and the sustainability of the settlers. It is worth noting that by the Early PPNB, cereals were already domesticated^36^. Within this context, the domestication of the faba bean represents an important additional step that settlers took to reduce the risk of famine.

## Conclusions

The present study provides new insights about the geographic area where the faba bean originated. The large stocks of seeds found in the coeval adjacent sites of Ahihud, Nahal Zippori 3 and Yiftah’el show that the faba bean was already domesticated in the 11^th^ millennium cal BP in the lower Galilee, Israel. Agronomic studies of the modern faba bean, show that cultivation alone does not improve the yield. Therefore only the selection of domestication traits, such as free-germination, could have maximized the agricultural output. The analysis of the Δ^13^C of the faba beans, which allows to identify the amount of water received by the plants during the period of growing, shows that the seeds from Ahihud were farmed under moister conditions compared to the seeds found in Yiftah’el. In the light of this evidence, we conclude that the intensive farming of the faba bean began in the 11^th^ millennium in the lower Galilee in a period of greater water availability and continued into the 10^th^ millennium, due to the ability of the local farmers to select seeds able to germinate under dryer conditions.

## Methods

### Charring experiments on modern material

Several studies have sought to replicate the condition under which archaeobotanical remains have been charred. Scholars found out that well preserved and undistorted archaeobotanical grain are more likely to have been heated at lower temperature (between 150 °C and 300 °C) in reducing conditions^37^,^38^,^39^,^40^. As results, we decided to conduct our experimental charring in reducing conditions at variable temperatures for different periods of time. For the experiment, 120 small faba beans (*Vicia faba* var*. minor*) were used because of the similarity in size and shape of this variety to the archaeological specimens. Length, breath and thickness of the seeds were measured using a Leica Image System Analysis (LAS V3.8) attached to a binocular microscope (Leica M80). Five groups, of 24 seeds each, were charred at 200 °C × 4 h, 200 °C × 12 h, 250 °C × 2 h, 250 °C × 4 h, 300 °C × 0.5 h in anoxic atmosphere in a muffle oven. Each seed was wrapped in aluminum foil. Measurements of length, breath and thickness were taken after the charring only on those seeds that were still whole (Supplementary Table S1). Additional 64 seeds were used to check the variation of Δ^13^C as result of charring. Previous studies had already showed that Δ^13^C of seeds did not change as effect of charring^12^,^41^, but none of these studies have ever tested the effect on faba beans. We therefore decided to test the variation of Δ^13^C on 32 seeds kept in reducing condition in a muffle oven at a temperature of 200 °C × 4 h, and on another 32 that were burned in the same condition but for a longer period of time (12 h) (Supplementary Table S2).

### Size analysis of archaeological material

469 seeds were selected among the remains collected in the archaeological contexts: the two storage pits found in Ahihud (L450_E14; L398_D13), the plaster floor in Nahal Zippori 3 (L273_C11), and the storage contexts from Yiftah’el (L5073_G18; L715_F40). The length, breadth and thickness were measured using a Leica Image System Analysis (LAS V3.8) attached to a binocular microscope (Leica M80) (Supplementary Table 3). Since experiment on modern material showed that breadth and thickness vary in unpredictable ways as result of charring, length was the only parameter used to compare the size of seeds coming from the different contexts.

A Z-Test at two row was used to prove that the average length of the populations were statistically different. P value < 0,001 was considered the threshold to accept this hypothesis (Supplementary Table S4).

### Radiocarbon dating of archaeological material

The samples were pre-treated, graphitized and measured by Accelerator Mass Spectrometry at D-REAMS Radiocarbon Laboratory of the Weizmann Institute of Science, Israel. The legumes (~30 mg of material) were cleaned using Acid-Base-Acid treatment as in Yitzhaq *et al*.^42^. The samples prepared for dating were combusted to CO_2_ in quartz tubes containing about 200 mg of copper oxide (Merck) and heated to 900 °C for 200 min. The CO_2_ was divided into 3 aliquots and each was reduced to graphite using cobalt (Fluka) (about 1 mg) as a catalyst and hydrogen at 700 °C for 20 hr. The ^14^C ages were calibrated to calendar years BP using the IntCal13 atmospheric curve^13^ using the software OxCal v 4.2.3^54^. Bayesian modeling was used to build the chronological sequence between the three sites according to the material culture remains. The legumes found in Ahihud are considered to be within the EPPNB and therefore are the earliest, while the material from Nahal Zippori 3 and Yiftha’el, both MPPNB, are younger. The dates of Ahihud start the model. Archaeologically, the storage places from Ahihud were not synchronous, therefore the ^14^C dates RTK 6866, 6875 and 6868 are set as a sequence within themselves. Finally, dates from Nahal Zippori 3 and Yiftha’el (RTK 6864,6865, 6892 and 6991) were added to the model in a phase, since there were no chronological differences among these two MPPNB sites (Supplementary Table S5).

### Stable carbon isotopes analysis of archaeological material

95 archaeological seeds were selected among the specimens found in the three sites. The seeds were purified from all contaminants using the same treatment reported for ^14^C samples. Analysis of the organic carbon by the dry combustion method was performed using an Elemental Analyzer (1112 Flash EA, Thermo-Finnigan) interfaced with an Isotope Ratio Mass Spectrometer (EA-IRMS Delta V Plus, Thermo Scientific), under the following conditions: the oxidation reactor was filled with chromic oxide over silvered cobaltous-cobaltic oxide and was maintained at 1020 °C. The reduction reactor filled with reduced copper wire was maintained at 650 °C. The chromatographic column for the gas separation was maintained at 50 °C. Helium carrier gas flow was 100 ml/min.

To account for changes in δ^13^C of atmospheric CO_2_ (δ^13^C_air_) during the two hundred year span between the sites, the Δ^13^C of the plant was calculated using the δ^13^C_air_ and the carbon isotope ratio of the plant (δ^13^C_plant_) as described by Farquhar *et al*.^43^.


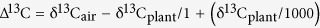


Where the δ^13^C_air_ was inferred by interpolating a range of data from ice-core records covering the whole Holocene (http://ftp.cmdl.noaa.gov/ccg/; http://web.udl.es/usuaris/x3845331/AIRCO2_LOESS.xls). The Δ^13^C was calculated for all the archaeological faba beans, using these updated estimates of past δ^13^C_air_^43^. The average value of Δ^13^C of each group as average value of 19 measurements. The higher is the value of the discriminant Δ^13^C the higher is the water input received by the faba bean during the period of growth. (Supplementary Table S6). A Z-Test at two row was used to prove that the average length of the populations were statistically different. P value < 0,001 was considered the threshold to accept this hypothesis (Supplementary Table S7).

## Additional Information

**How to cite this article**: Caracuta, V. *et al*. The onset of faba bean farming in the Southern Levant. *Sci. Rep*. **5**, 14370; doi: 10.1038/srep14370 (2015).

## Supplementary Material

Supplementary Information

## Figures and Tables

**Figure 1:**
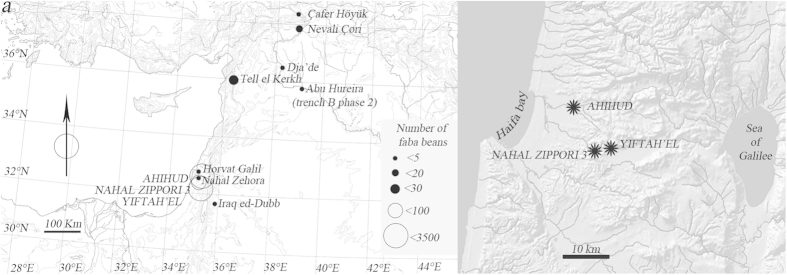
Faba bean remains in the prehistoric sites of the Levant. (**a**) Map showing all the Neolithic sites where the faba bean was found: Çafer Höyük^45^, Nevali Cori^46^, Abu Hureyra^47^, Dja’de^48^ Tell el Kerkh^49^, Iraq ed-Dubb^50^, Nahal Zehora^51^, Horvat Galil^52^, Yiftah’el^35^ (and this work), Nahal Zippori 3^53^ and Ahihud (this work) (the map used is courtesy of the Oriental Institute of the University of Chicago, Photo: Anna Ressman’) (**b**) Detailed map showing the locations of the sites presented in this study - Ahihud, Nahal Zippori 3 and Yiftah’el (the map used is courtesy of the Israel Antiquities Authority, Map: MichalBirkenfeld).

**Figure 2:**
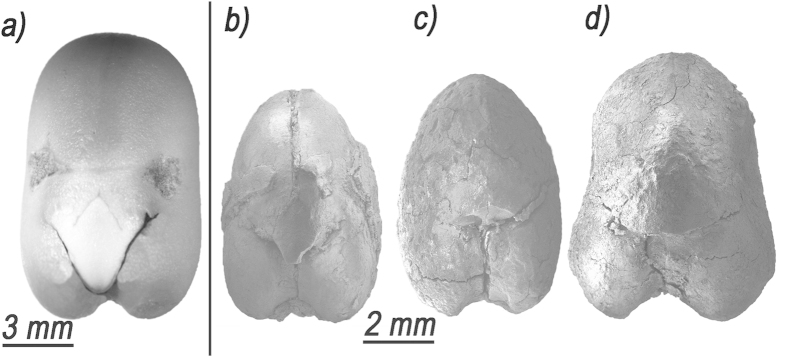
Archaeological and modern faba beans (**a**) SEM images of modern *Vicia faba* var. minor. (**b**) *Vicia faba* L. from Yiftah’el. (**c**) Nahal Zippori 3. (**d**) Ahihud.
