# Supplementary material for: The onset of faba bean farming in the Southern Levant
Source: Sci Rep. 2015 Oct 13;5:14370. doi: 10.1038/srep14370 (PMC4602238; doi:10.1038/srep14370)
Supplement: Supplementary Information [file srep14370-s1.doc]

**The onset of faba bean farming in the Southern Levant.**

Valentina Caracuta, Omry Barzilai, Hamudi Khalaily, Ianir Milevski, Yitzhak Paz, Jacob Vardi, Lior Regev and Elisabetta Boaretto

**Supplementary information**

**Supplementary Table S1| The effect of differential charring on length, breadth and thickness (mm) on 120 seeds of modern *Vicia faba* var. *minor*.** When a seed was only partially preserved and one of the dimensions could not be accurately measured the data was reported as (\).

|  | Fresh | | | After charring (200°x4h) | | |  | Fresh | | | After charring (200°x12h) | | |
| --- | --- | --- | --- | --- | --- | --- | --- | --- | --- | --- | --- | --- | --- |
| **ID sample** | Length | Breadth | Thickness | Length | Breadth | Thickness | **ID sample** | Length | Breadth | Thickness | Length | Breadth | Thickness |
| **1** | 10,14 | 8,08 | 7,66 | 8,97 | 6,92 | 6,75 | **25** | 7,39 | 7,10 | 5,75 | 6,63 | 5,81 | 5,16 |
| **2** | 8,55 | 7,23 | 6,77 | 7,21 | 6,05 | 5,69 | **26** | 9,47 | 6,17 | 5,93 | 8,21 | 4,93 | 5,04 |
| **3** | 8,80 | 7,26 | 6,32 | 7,57 | 5,82 | 5,42 | **27** | 8,41 | 6,73 | 5,84 | 7,33 | 5,99 | 5,80 |
| **4** | 9,25 | 8,71 | 7,69 | 8,09 | 7,22 | 6,73 | **28** | 10,61 | 7,95 | 7,14 | 8,88 | 6,44 | 5,98 |
| **5** | 8,42 | 6,75 | 6,6 | 7,05 | 5,78 | 5,57 | **29** | 7,93 | 6,86 | 6,35 | 6,67 | 5,65 | 5,49 |
| **6** | 9,13 | 6,77 | 5,58 | 7,88 | 5,43 | 4,93 | **30** | 10,86 | 7,82 | 6,47 | 9,12 | 6,51 | 5,83 |
| **7** | 10,15 | 7,54 | 7,00 | 8,61 | 6,18 | 5,89 | **31** | 8,23 | 6,55 | 5,85 | 6,98 | 5,50 | 5,26 |
| **8** | 10,23 | 8,19 | 7,32 | 8,44 | 6,38 | 6,10 | **32** | 9,34 | 7,14 | 6,81 | 7,48 | 5,39 | 5,55 |
| **9** | 9,00 | 6,76 | 6,59 | 7,26 | 4,93 | 4,90 | **33** | 10,94 | 7,35 | 6,89 | 8,98 | 5,99 | 5,76 |
| **10** | 8,85 | 6,85 | 5,86 | 7,08 | 5,28 | 4,65 | **34** | 9,15 | 7,22 | 6,55 | 7,72 | 5,70 | 5,30 |
| **11** | 10,91 | 7,47 | 6,57 | 9,13 | 6,10 | 5,42 | **35** | 9,75 | 7,30 | 6,66 | 7,97 | 5,71 | 5,37 |
| **12** | 9,76 | 7,04 | 6,62 | 8,29 | 5,81 | 5,75 | **36** | 10,99 | 8,48 | 7,12 | 9,12 | 6,76 | 6,07 |
| **13** | 8,74 | 6,42 | 6,32 | 7,16 | 5,19 | 5,22 | **37** | 10,18 | 7,30 | 6,79 | 8,36 | 5,79 | 5,59 |
| **14** | 9,53 | 7,54 | 6,75 | 7,76 | 6,04 | 5,63 | **38** | 9,00 | 6,65 | 6,22 | 7,65 | 5,60 | 5,23 |
| **15** | 8,75 | 7,29 | 7,31 | 7,09 | 5,67 | 5,84 | **39** | 9,23 | 7,16 | 6,70 | 7,25 | 5,80 | 5,52 |
| **16** | 9,93 | 7,01 | 6,25 | 8,15 | 5,45 | 5,26 | **40** | 10,09 | 7,63 | 6,55 | 8,25 | 6,22 | 5,98 |
| **17** | 8,48 | 7,09 | 6,42 | 6,96 | 5,46 | 5,23 | **41** | 8,71 | 6,34 | 5,93 | 7,04 | 4,93 | 4,89 |
| **18** | 10,29 | 7,17 | 6,27 | 8,58 | 5,74 | 4,97 | **42** | 8,87 | 6,61 | 6,01 | 7,19 | 5,23 | 4,97 |
| **19** | 10,17 | 8,09 | 7,37 | 8,64 | 6,68 | 6,19 | **43** | 10,76 | 7,59 | 6,80 | 9,31 | 6,22 | 6,33 |
| **20** | 11,02 | 7,56 | 7,21 | 9,18 | 6,39 | 6,15 | **44** | 9,64 | 7,25 | 6,30 | 8,05 | 5,86 | 5,46 |
| **21** | 7,10 | 6,38 | 5,67 | 6,17 | 5,22 | 4,94 | **45** | 8,77 | 7,06 | 6,24 | 7,25 | 5,67 | 5,49 |
| **22** | 10,57 | 7,54 | 6,99 | 8,74 | 6,08 | 5,99 | **46** | 8,75 | 6,32 | 5,79 | 7,09 | 5,03 | 4,72 |
| **23** | 9,90 | 7,21 | 6,92 | 8,31 | 5,74 | 5,92 | **47** | 8,66 | 6,88 | 6,43 | 7,37 | 5,55 | 5,56 |
| **24** | 9,38 | 7,31 | 6,22 | 8,15 | 6,00 | 5,43 | **48** | 9,88 | 6,84 | 6,19 | 8,32 | 5,67 | 5,42 |
| **Average** | 9,46 | 7,30 | 6,68 | 7,94 | 5,90 | 5,61 | **Average** | 9,40 | 7,10 | 6,39 | 7,84 | 5,75 | 5,49 |
| **SD (±)** | 0,92 | 0,56 | 0,58 | 0,80 | 0,56 | 0,55 | **SD (±)** | 1,00 | 0,55 | 0,42 | 0,82 | 0,47 | 0,39 |

|  | Fresh | | | After charring (250°x4h) | | |  | Fresh | | | After charring (250°x12h) | | |
| --- | --- | --- | --- | --- | --- | --- | --- | --- | --- | --- | --- | --- | --- |
| **ID sample** | Length | Breadth | Thickness | Length | Breadth | Thickness | **ID sample** | Length | Breadth | Thickness | Lenght | Breadth | Thickness |
| **49** | 10,57 | 7,70 | 6,09 | 9,27 | 6,16 | 5,46 | **73** | 9,21 | 7,34 | 7,13 | 8,15 | 6,22 | 6,61 |
| **50** | 11,20 | 8,00 | 6,50 | 9,32 | 6,30 | 5,21 | **74** | 9,29 | 6,71 | 6,35 | 8,10 | 5,39 | 5,73 |
| **51** | 9,71 | 7,33 | 6,78 | 8,47 | 6,13 | 6,16 | **75** | 10,00 | 8,02 | 6,94 | 8,38 | 6,10 | 5,88 |
| **52** | 10,20 | 7,85 | 6,94 | 8,81 | 6,21 | 5,85 | **76** | 9,82 | 7,17 | 6,53 | 8,72 | 6,37 | 6,25 |
| **53** | 10,17 | 7,23 | 6,19 | 9,01 | 5,95 | 5,48 | **77** | 9,09 | 6,87 | 5,96 | 8,43 | 5,70 | 5,47 |
| **54** | 9,08 | 6,77 | 6,09 | 7,69 | 5,98 | 5,8 | **78** | 11,06 | 7,79 | 6,38 | 9,21 | 6,14 | 5,39 |
| **55** | 9,23 | 7,01 | 6,43 | 7,67 | 5,39 | 5,47 | **79** | 9,45 | 7,82 | 6,56 | 7,64 | 5,88 | 5,46 |
| **56** | 10,51 | 7,39 | 6,65 | 9,02 | 6,04 | 5,81 | **80** | 9,25 | 6,99 | 6,63 | 8,60 | 6,25 | 6,08 |
| **57** | 10,12 | 7,61 | 6,68 | \ | \ | \ | **81** | 10,00 | 7,86 | 6,63 | \ | \ | \ |
| **58** | 10,22 | 7,03 | 6,58 | \ | \ | \ | **82** | 9,35 | 6,92 | 6,86 | \ | \ | \ |
| **59** | 9,70 | 7,57 | 6,97 | \ | \ | \ | **83** | 9,40 | 7,30 | 6,48 | \ | \ | \ |
| **60** | 10,35 | 7,13 | 6,76 | \ | \ | \ | **84** | 10,38 | 7,88 | 6,97 | \ | \ | \ |
| **61** | 8,74 | 7,31 | 6,35 | \ | \ | \ | **85** | 8,78 | 6,63 | 6,30 | \ | \ | \ |
| **62** | 9,83 | 7,29 | 6,45 | 8,05 | 5,8 | 5,31 | **86** | 10,12 | 7,48 | 6,97 | \ | \ | \ |
| **63** | 8,88 | 7,19 | 6,69 | \ | \ | \ | **87** | 9,16 | 7,09 | 6,82 | \ | \ | \ |
| **64** | 9,41 | 6,77 | 6,32 | \ | \ | \ | **88** | 8,84 | 7,39 | 6,95 | \ | \ | \ |
| **65** | 8,88 | 6,41 | 5,98 | 7,29 | 5,25 | 5,07 | **89** | 9,40 | 6,91 | 6,54 | \ | \ | \ |
| **66** | 9,10 | 6,97 | 6,75 | 7,61 | 6,30 | 5,80 | **90** | 8,37 | 6,38 | 5,99 | 7,58 | 5,84 | 5,75 |
| **67** | 8,85 | 6,53 | 5,85 | 7,79 | 5,46 | 5,20 | **91** | 7,66 | 6,26 | 5,49 | 6,69 | 5,15 | 4,69 |
| **68** | 8,36 | 6,20 | 5,66 | 7,42 | 5,75 | 5,21 | **92** | 8,89 | 6,86 | 6,04 | 8,52 | 5,96 | 5,86 |
| **69** | 9,90 | 7,59 | 7,02 | 8,79 | 7,18 | 6,81 | **93** | 8,92 | 6,35 | 5,55 | 7,81 | 5,54 | 5,23 |
| **70** | 8,80 | 6,66 | 6,06 | \ | \ | \ | **94** | 7,19 | 6,03 | 5,88 | 6,32 | 5,96 | 5,36 |
| **71** | 9,02 | 7,08 | 6,59 | \ | \ | \ | **95** | 9,25 | 7,64 | 6,65 | 8,19 | 6,21 | 5,96 |
| **72** | 10,22 | 7,01 | 6,25 | 8,53 | 5,73 | 5,00 | **96** | 9,95 | 6,89 | 6,29 | 8,78 | 5,88 | 5,77 |
| **Average** | 9,63 | 7,15 | 6,44 | 8,32 | 5,98 | 5,58 | **Average** | 9,28 | 7,11 | 6,45 | 8,07 | 5,91 | 5,70 |
| **SD (±)** | 0,73 | 0,45 | 0,37 | 0,70 | 0,47 | 0,48 | **SD (±)** | 0,82 | 0,56 | 0,45 | 0,78 | 0,34 | 0,46 |

**Continuation from Supplementary Table S1**

|  | Fresh | | | After charring (300°x0.5h) | | |
| --- | --- | --- | --- | --- | --- | --- |
| **ID Sample** | Length | Breadth | Thickness | Length | Breadth | Thickness |
| **97** | 9,94 | 7,22 | 6,37 | \ | \ | \ |
| **98** | 10,05 | 7,10 | 6,03 | \ | \ | \ |
| **99** | 9,95 | 8,03 | 7,10 | \ | \ | \ |
| **100** | 8,35 | 6,38 | 6,13 | \ | \ | \ |
| **101** | 9,38 | 7,01 | 6,44 | \ | \ | \ |
| **102** | 10,17 | 7,29 | 6,23 | \ | \ | \ |
| **103** | 8,45 | 6,84 | 6,17 | \ | \ | \ |
| **104** | 10,83 | 6,87 | 5,81 | \ | \ | \ |
| **105** | 8,98 | 7,45 | 6,48 | \ | \ | \ |
| **106** | 10,65 | 8,03 | 6,30 | \ | \ | \ |
| **107** | 10,27 | 7,54 | 6,65 | \ | \ | \ |
| **108** | 9,29 | 7,15 | 6,24 | \ | \ | \ |
| **109** | 10,47 | 6,67 | 6,12 | \ | \ | \ |
| **110** | 8,51 | 6,74 | 6,34 | \ | \ | \ |
| **111** | 9,27 | 7,68 | 7,09 | \ | \ | \ |
| **112** | 9,92 | 7,69 | 6,74 | \ | \ | \ |
| **113** | 9,33 | 7,15 | 6,52 | \ | \ | \ |
| **114** | 8,51 | 7,62 | 8,36 | \ | \ | \ |
| **115** | 10,29 | 7,34 | 6,69 | \ | \ | \ |
| **116** | 10,27 | 7,98 | 7,04 | \ | \ | \ |
| **117** | 10,49 | 7,98 | 6,64 | \ | \ | \ |
| **118** | 9,97 | 7,81 | 7,21 | \ | \ | \ |
| **119** | 8,26 | 6,79 | 6,50 | \ | \ | \ |
| **120** | 9,12 | 6,27 | 5,75 | \ | \ | \ |
| **Average** | 9,61 | 7,28 | 6,54 | \ | \ | \ |
| **SD (±)** | 0,80 | 0,52 | 0,55 | \ | \ | \ |

**Supplementary Table S2| The effect of differential charring on the δ^13^C (‰) (and related Δ^13^C) on 64 seeds of modern *Vicia faba* var. *minor*.** The Δ^13^C was calculated using the δ^13^C_air_= -8 ‰.

| Fresh | | | | After charring( 200°x4h) | | |  | Fresh | | | After charring (200°x12h) | | |
| --- | --- | --- | --- | --- | --- | --- | --- | --- | --- | --- | --- | --- | --- |
| **ID sample** | δ^13^C | SD (±) | Δ^13^C | δ^13^C | SD (±) | Δ^13^C | **ID sample** | δ^13^C | SD (±) | Δ^13^C | δ^13^C (‰) | SD (±) | Δ^13^C |
| **1** | -27,10 | 0,20 | 19,65 | -27,20 | 0,10 | 19,75 | **33** | -26,10 | 0,30 | 18,60 | -25,70 | 0,40 | 18,20 |
| **2** | -27,30 | 0,40 | 19,85 | -27,60 | 0,50 | 20,15 | **34** | -27,50 | 0,30 | 20,05 | -27,50 | 0,10 | 20,05 |
| **3** | -26,60 | 0,30 | 19,10 | -26,20 | 0,40 | 18,70 | **35** | -26,90 | 0,20 | 19,40 | -26,90 | 0,20 | 19,40 |
| **4** | -27,20 | 0,10 | 19,75 | -27,00 | 0,70 | 19,55 | **36** | -26,00 | 0,20 | 18,50 | -26,10 | 0,10 | 18,60 |
| **5** | -27,30 | 0,20 | 19,85 | -27,40 | 0,20 | 19,95 | **37** | -27,30 | 0,10 | 19,85 | -26,80 | 0,60 | 19,30 |
| **6** | -26,40 | 0,10 | 18,90 | -26,30 | 0,01 | 18,80 | **38** | -27,10 | 0,10 | 19,65 | -26,90 | 0,10 | 19,40 |
| **7** | -26,80 | 0,50 | 19,30 | -27,70 | 0,20 | 20,25 | **39** | -26,20 | 0,10 | 18,70 | -26,60 | 0,10 | 19,10 |
| **8** | -26,60 | 0,20 | 19,10 | -27,00 | 0,01 | 19,55 | **40** | -24,50 | 0,10 | 16,90 | -24,50 | 0,30 | 16,90 |
| **9** | -27,50 | 0,10 | 20,05 | -26,80 | 0,10 | 19,30 | **41** | -26,70 | 0,10 | 19,20 | -26,90 | 0,10 | 19,40 |
| **10** | -26,70 | 0,09 | 19,20 | -26,00 | 0,24 | 18,50 | **42** | -27,20 | 0,10 | 19,75 | -27,30 | 0,10 | 19,90 |
| **11** | -25,60 | 0,09 | 18,05 | -25,30 | 0,39 | 17,75 | **43** | -28,40 | 0,10 | 21,00 | -28,40 | 0,17 | 21,00 |
| **12** | -25,20 | 0,22 | 17,65 | -25,30 | 0,16 | 17,75 | **44** | -26,40 | 0,14 | 18,90 | -26,10 | 0,02 | 18,60 |
| **13** | -26,60 | 0,01 | 19,10 | -26,90 | 0,29 | 19,40 | **45** | -26,90 | 0,08 | 19,40 | -26,40 | 0,14 | 18,90 |
| **14** | -27,00 | 0,02 | 19,55 | -26,80 | 0,05 | 19,30 | **46** | -26,80 | 0,13 | 19,30 | -26,20 | 0,01 | 18,70 |
| **15** | -27,10 | 0,06 | 19,63 | -26,80 | 0,15 | 19,32 | **47** | -26,80 | 0,04 | 19,32 | -26,70 | 0,08 | 19,21 |
| **16** | -27,20 | 0,05 | 19,75 | -26,50 | 0,64 | 19,00 | **48** | -26,70 | 0,09 | 19,20 | -26,60 | 0,06 | 19,10 |
| **17** | -28,00 | 0,02 | 20,6 | -27,30 | 0,54 | 19,85 | **49** | -26,20 | 0,03 | 18,70 | -25,90 | 0,16 | 18,40 |
| **18** | -26,90 | 0,04 | 19,40 | -26,50 | 0,53 | 19,00 | **50** | -28,10 | 0,06 | 20,70 | -27,70 | 0,09 | 20,25 |
| **19** | -26,80 | 0,04 | 19,30 | -26,70 | 0,06 | 19,20 | **51** | -29,00 | 0,03 | 21,65 | -28,90 | 0,02 | 21,50 |
| **20** | -28,10 | 0,20 | 20,70 | -28,00 | 0,03 | 20,60 | **52** | -28,10 | 0,02 | 20,70 | -27,80 | 0,28 | 20,40 |
| **21** | -26,80 | 0,03 | 19,30 | -25,90 | 0,19 | 18,40 | **53** | -28,40 | 0,01 | 21,00 | -28,30 | 0,14 | 20,90 |
| **22** | -26,30 | 0,02 | 18,80 | -26,00 | 0,01 | 18,50 | **54** | -26,70 | 0,70 | 19,20 | -26,10 | 0,70 | 18,60 |
| **23** | -28,40 | 0,04 | 21,00 | -28,00 | 0,11 | 20,60 | **55** | -27,80 | 0,03 | 20,40 | -26,70 | 0,34 | 19,20 |
| **24** | -25,90 | 0,06 | 18,40 | -25,50 | 0,34 | 18,00 | **56** | -27,90 | 0,01 | 20,50 | -27,50 | 0,02 | 20,05 |
| **25** | -26,50 | 0,18 | 19,00 | -26,30 | 0,38 | 18,80 | **57** | -26,80 | 0,78 | 19,30 | -26,20 | 0,06 | 18,70 |
| **26** | -28,70 | 0,09 | 21,30 | -28,60 | 0,18 | 21,20 | **58** | -28,20 | 0,02 | 20,80 | -28,50 | 0,07 | 21,10 |
| **27** | -26,20 | 0,01 | 18,70 | -26,00 | 0,11 | 18,50 | **59** | -26,20 | 0,03 | 18,70 | -24,80 | 0,11 | 17,25 |
| **28** | -24,30 | 0,13 | 16,70 | -25,40 | 0,10 | 17,85 | **60** | -28,00 | 0,09 | 20,60 | -27,60 | 0,35 | 20,15 |
| **29** | -25,40 | 0,10 | 17,85 | -24,80 | 0,48 | 17,25 | **61** | -28,00 | 0,04 | 20,60 | -27,60 | 0,18 | 20,15 |
| **30** | -27,00 | 0,77 | 19,55 | -26,30 | 0,19 | 18,80 | **62** | -27,50 | 0,07 | 20,05 | -27,50 | 0,02 | 20,05 |
| **31** | -27,10 | 0,46 | 19,65 | -27,30 | 0,09 | 19,85 | **63** | -25,50 | 0,11 | 18,00 | -25,90 | 1,18 | 18,40 |
| **32** | -27,80 | 0,08 | 20,40 | -27,50 | 0,25 | 20,05 | **64** | -26,30 | 0,22 | 18,80 | -25,00 | 0,21 | 17,45 |

**Supplementary Table S3| Length, breadth and thickness (mm) of 469 archaeological *Vicia faba* L.** When a seed was only partially preserved and one of the dimensions could not be accurately measured the data was reported as (\).

|  | Ahihud L450_E14 | | | | | | | | | | |
| --- | --- | --- | --- | --- | --- | --- | --- | --- | --- | --- | --- |
| **ID sample** | Length | Breadth | Thickness | **ID sample** | Length | Breadth | Thickness | **ID sample** | Length | Breadth | Thickness |
| **1** | 5,94 | 4,91 | 4,6 | **44** | 5,78 | 4,64 | 4,13 | **87** | 6,56 | 5,27 | 4,47 |
| **2** | 6,59 | 5,08 | 4,49 | **45** | 5,93 | 4,56 | 3,97 | **88** | 6,30 | 4,72 | 4,17 |
| **3** | 5,84 | 4,06 | 4,03 | **46** | 6,36 | 4,92 | 4,23 | **89** | 5,95 | 4,62 | 3,93 |
| **4** | 5,86 | 4,45 | 4,53 | **47** | 6,3 | 4,73 | 3,46 | **90** | 6,01 | 4,58 | 4,07 |
| **5** | 5,27 | 4,90 | 4,50 | **48** | 6,06 | 5,03 | 4,58 | **91** | 6,11 | 5,03 | 3,95 |
| **6** | 5,17 | 4,42 | 4,19 | **49** | 6,62 | 4,57 | 4,35 | **92** | 6,02 | 4,80 | 4,63 |
| **7** | 6,28 | 4,87 | 3,91 | **50** | 6,24 | 4,62 | 4,53 | **93** | 6,67 | 4,91 | 4,62 |
| **8** | 5,29 | 4,10 | 3,70 | **51** | 6,43 | 5,45 | 4,23 | **94** | 6,23 | 4,90 | 4,16 |
| **9** | 5,98 | 5,13 | 4,36 | **52** | 6,75 | 4,90 | 4,03 | **95** | 5,90 | 4,88 | 4,80 |
| **10** | 5,32 | 4,18 | 3,48 | **53** | 6,43 | 4,89 | 4,27 | **96** | 6,28 | 4,80 | 4,44 |
| **11** | 6,26 | 4,68 | 4,78 | **54** | 5,97 | 5,33 | 4,54 | **97** | 5,78 | 5,03 | 4,52 |
| **12** | 6,42 | 4,98 | 4,94 | **55** | 6,24 | 4,82 | 4,40 | **98** | 6,24 | 4,95 | 4,41 |
| **13** | 6,01 | 4,82 | 4,58 | **56** | 5,96 | 4,92 | 4,85 | **99** | 5,89 | 3,89 | 4,22 |
| **14** | 5,63 | 4,60 | 4,40 | **57** | 5,94 | 4,75 | 4,34 | **100** | 6,57 | 4,88 | 4,34 |
| **15** | 6,06 | 5,02 | 4,70 | **58** | 5,47 | 4,50 | 4,72 | **101** | 6,86 | 5,70 | 4,43 |
| **16** | 6,22 | 5,36 | 5,03 | **59** | 6,47 | 4,81 | 4,53 | **102** | 6,24 | 5,17 | 4,69 |
| **17** | 6,41 | 5,46 | 5,19 | **60** | 6,25 | 5,11 | 4,56 | **103** | 6,60 | 5,50 | 4,11 |
| **18** | 5,80 | 4,89 | 4,33 | **61** | 6,13 | 4,48 | \ | **104** | 6,78 | 4,58 | 5,01 |
| **19** | 6,76 | 5,02 | 4,39 | **62** | 6,08 | 4,78 | 4,39 | **105** | 6,63 | 4,77 | 4,76 |
| **20** | 6,33 | 5,76 | 4,83 | **63** | 6,88 | 5,39 | 4,32 | **106** | 7,22 | 4,93 | 4,12 |
| **21** | 5,73 | 4,87 | 4,26 | **64** | 5,59 | 4,95 | 4,37 | **107** | 6,07 | 4,76 | 3,94 |
| **22** | 5,97 | 5,04 | 4,73 | **65** | 5,98 | 5,02 | 4,78 | **108** | 5,66 | 5,03 | 4,34 |
| **23** | 6,92 | 5,19 | 4,88 | **66** | 5,83 | 4,65 | 4,16 | **109** | 5,73 | 5,15 | 4,01 |
| **24** | 6,55 | 4,86 | 4,66 | **67** | 6,16 | 5,42 | 5,18 | **110** | 5,89 | 5,27 | 4,91 |
| **25** | 6,04 | 4,60 | 4,60 | **68** | 5,40 | 4,69 | 4,59 | **111** | 6,34 | 4,70 | 4,34 |
| **26** | 7,17 | 6,12 | 5,10 | **69** | 7,23 | 5,80 | 4,32 | **112** | 5,97 | 5,32 | 4,35 |
| **27** | 6,30 | 5,19 | 4,78 | **70** | 6,04 | 4,92 | 4,57 | **113** | 6,22 | 4,72 | 4,71 |
| **28** | 5,82 | 4,58 | 4,16 | **71** | 6,68 | 5,86 | 5,18 | **114** | 6,83 | 5,1 | 4,60 |
| **29** | 6,71 | 5,00 | 4,88 | **72** | 6,53 | 5,55 | 4,91 | **115** | 6,26 | 5,95 | 4,77 |
| **30** | 6,13 | 5,61 | 5,00 | **73** | 6,21 | 5,59 | 5,10 | **116** | 6,34 | 5,15 | 4,53 |
| **31** | 6,11 | 5,08 | 4,61 | **74** | 6,09 | 4,99 | 4,11 | **117** | 6,16 | 4,87 | 4,50 |
| **32** | 6,31 | 5,39 | 4,42 | **75** | 6,12 | 4,43 | 4,31 | **118** | 6,82 | 5,47 | 5,41 |
| **33** | 5,79 | 4,81 | 4,96 | **76** | 6,29 | 4,94 | 4,47 | **119** | 6,54 | 5,41 | 5,09 |
| **34** | 5,84 | 4,70 | 4,08 | **77** | 6,74 | 5,67 | 5,18 | **120** | 5,93 | 4,75 | 4,40 |
| **35** | 6,00 | 4,95 | \ | **78** | 5,42 | 4,62 | 3,96 | **121** | 5,85 | 4,87 | 4,08 |
| **36** | 5,45 | 4,21 | \ | **79** | 6,01 | 5,34 | 4,93 | **122** | 6,03 | 5,24 | 4,21 |
| **37** | 5,30 | 4,19 | \ | **80** | 6,14 | 4,90 | 4,28 | **123** | 6,10 | 4,83 | 4,29 |
| **38** | 5,29 | 4,65 | \ | **81** | 7,14 | 6,04 | 5,62 | **124** | 5,90 | 4,49 | 4,41 |
| **39** | 4,40 | 3,56 | \ | **82** | 6,40 | 5,18 | 5,12 | **125** | 5,70 | 4,55 | 4,41 |
| **40** | 5,28 | 3,97 | \ | **83** | 6,42 | 4,63 | 4,35 | **126** | 6,31 | 5,25 | 4,57 |
| **41** | 6,19 | 5,07 | 4,59 | **84** | 5,34 | 4,25 | 4,09 | **127** | 5,73 | 5,18 | 4,48 |
| **42** | 6,90 | 5,10 | 4,90 | **85** | 5,60 | 4,80 | 4,44 | **128** | 6,12 | 4,92 | 4,56 |
| **43** | 5,87 | 4,56 | 4,50 | **86** | 5,64 | 4,47 | 4,36 | **Average** | 6,13 | 4,93 | 4,49 |
|  |  |  |  |  |  |  |  | **SD(±)** | 0,46 | 0,43 | 0,37 |

**Continuation of Supplementary Table S3**

| Ahihud L398_D13 | | | | | | | | Nahal Zippori 3 L273_C11 | | | |
| --- | --- | --- | --- | --- | --- | --- | --- | --- | --- | --- | --- |
| **ID sample** | Length | Breadth | Thickness | ID sample | Length | Breadth | Thickness | **ID sample** | Length | Breadth | Thickness |
| **129** | 6,88 | 5,87 | 4,54 | **168** | 5,44 | 4,03 | 3,85 | **206** | 5,61 | 4,50 | 4,55 |
| **130** | 7,15 | 5,13 | 5,26 | **169** | 5,63 | 5,43 | 4,32 | **207** | 5,50 | 4,74 | 3,80 |
| **131** | 6,65 | 5,16 | 4,18 | **170** | 5,71 | 4,65 | 4,12 | **208** | 5,69 | 4,70 | 3,99 |
| **132** | 6,83 | 5,03 | 4,70 | **171** | 5,48 | 4,25 | 3,92 | **209** | 5,44 | 4,63 | 4,44 |
| **133** | 6,00 | 4,65 | 4,47 | **172** | 6,28 | 4,49 | 4,76 | **210** | 4,70 | 4,24 | 3,75 |
| **134** | 6,23 | 4,47 | 4,35 | **173** | 6,07 | 5,00 | 4,74 | **211** | 5,45 | 4,62 | \ |
| **135** | 7,25 | 5,49 | 5,44 | **174** | 5,64 | 4,78 | 4,30 | **212** | 5,33 | 4,54 | \ |
| **136** | 6,96 | 5,92 | 4,96 | **175** | 5,94 | 4,36 | 4,15 | **213** | 6,98 | 5,31 | 4,04 |
| **137** | 6,88 | 5,65 | 4,90 | **176** | 5,83 | 4,66 | 4,40 | **214** | 4,87 | 4,23 | 4,54 |
| **138** | 6,74 | 4,33 | 4,54 | **177** | 6,23 | 4,80 | 4,12 | **215** | 4,63 | 4,06 | 3,45 |
| **139** | 5,56 | 4,95 | 4,40 | **178** | 5,64 | 5,00 | 4,34 | **216** | 4,76 | 4,41 | 3,53 |
| **140** | 5,55 | 4,17 | 3,78 | **179** | 5,36 | 3,97 | 3,73 | **217** | 4,50 | 3,82 | \ |
| **141** | 6,73 | 5,56 | 5,14 | **180** | 6,14 | 4,98 | 4,59 | **218** | 5,25 | 4,51 | \ |
| **142** | 6,19 | 4,80 | 4,70 | **181** | 5,34 | 4,06 | 3,76 | **219** | 4,21 | \ | \ |
| **143** | 4,80 | 4,08 | 4,08 | **182** | 5,43 | 4,60 | 4,06 | **220** | 4,61 | 4,25 | \ |
| **144** | 5,59 | 4,23 | 2,43 | **183** | 5,39 | 4,82 | 3,58 | **221** | 5,75 | 4,91 | \ |
| **145** | 5,55 | 4,67 | 4,04 | **184** | 5,18 | 4,44 | 4,43 | **222** | 5,29 | 4,51 | 3,60 |
| **146** | 5,49 | 4,50 | 4,17 | **185** | 5,03 | 3,82 | 3,76 | **223** | 5,65 | 4,55 | 3,87 |
| **147** | 6,33 | 5,60 | 5,47 | **186** | 6,11 | 4,15 | \ | **224** | 4,79 | 4,40 | 4,14 |
| **148** | 6,35 | 4,03 | 4,38 | **187** | 5,26 | 4,07 | 3,73 | **225** | 5,21 | 4,41 | 3,95 |
| **149** | 5,45 | 3,98 | 3,60 | **188** | 5,23 | 4,82 | 3,41 | **226** | 5,33 | 4,68 | 3,91 |
| **150** | 5,53 | 4,15 | 3,92 | **189** | 5,42 | 4,59 | \ | **227** | 5,32 | 4,52 | 3,63 |
| **151** | 6,20 | 4,55 | 4,72 | **190** | 6,17 | 4,76 | 4,14 | **228** | 4,80 | 4,21 | 3,63 |
| **152** | 6,65 | 5,21 | 4,49 | **191** | 5,89 | 4,46 | 4,31 | **229** | 4,94 | 4,45 | 3,72 |
| **153** | 6,96 | 5,27 | 4,96 | **192** | 6,43 | 5,17 | 4,39 | **230** | 6,17 | \ | 4,19 |
| **154** | 6,88 | 5,97 | 4,74 | **193** | 6,20 | 4,48 | 4,30 | **231** | 4,86 | 4,05 | 4,03 |
| **155** | 5,52 | 4,74 | 3,98 | **194** | 5,24 | 4,28 | 4,55 | **232** | 5,28 | 4,53 | 3,74 |
| **156** | 6,02 | 5,13 | 4,69 | **195** | 5,73 | 4,75 | 3,77 | **233** | 5,63 | 4,32 | \ |
| **157** | 5,58 | 5,63 | 2,24 | **196** | 5,61 | 4,52 | 4,24 | **234** | 5,30 | 4,38 | \ |
| **158** | 5,64 | 4,19 | 3,72 | **197** | 5,63 | 4,21 | 4,13 | **235** | 5,02 | 4,56 | 3,80 |
| **159** | 5,83 | 4,60 | 4,56 | **198** | 5,32 | 4,63 | 4,52 | **236** | 6,04 | 5,44 | \ |
| **160** | 6,26 | 4,85 | 4,29 | **199** | 6,10 | 4,74 | \ | **237** | 5,23 | 4,36 | \ |
| **161** | 6,20 | 4,84 | 4,48 | **200** | 5,74 | 4,86 | 4,01 | **238** | 4,65 | 4,01 | 3,89 |
| **162** | 5,83 | 4,81 | 4,38 | **201** | 5,34 | 4,36 | 4,22 | **239** | 5,02 | 4,46 | 4,10 |
| **163** | 5,85 | 4,78 | 3,96 | **202** | 5,12 | 4,86 | 3,94 | **240** | 5,32 | \ | 3,97 |
| **164** | 6,06 | 5,19 | 4,34 | **203** | 5,58 | 4,53 | 3,48 | **241** | 4,99 | 4,3 | 3,62 |
| **165** | 5,70 | 4,90 | 4,18 | **204** | 5,58 | 4,10 | \ |  |  |  |  |
| **166** | 6,18 | 4,73 | 4,27 | **205** | 5,15 | 4,30 | 3,54 |  |  |  |  |
| **167** | 5,36 | 4,49 | 3,89 | **Average** | 5,9 | 4,71 | 4,23 | **Average** | 5,23 | 4,47 | 3,92 |
|  |  |  |  | **SD(±)** | 0,55 | 0,49 | 0,54 | **SD(±)** | 0,53 | 0,32 | 0,30 |

**Continuation of Supplementary Table S3**

|  | Yiftah'el L5073_G18 | | | | | | | | | | |
| --- | --- | --- | --- | --- | --- | --- | --- | --- | --- | --- | --- |
| **ID sample** | Length | Breadth | Thickness | **ID sample** | Length | Breadth | Thickness | **ID sample** | Length | Breadth | Thickness |
| **242** | 4,81 | 4,05 | 4,29 | **285** | 5,14 | 4,51 | 4,26 | **328** | 4,78 | 4,13 | 3,23 |
| **243** | 5,04 | 4,51 | 4,16 | **286** | 5,36 | 4,51 | 4,11 | **329** | 4,65 | 4,27 | 4,24 |
| **244** | 5,46 | 4,94 | 4,50 | **287** | 5,07 | 4,74 | 4,33 | **330** | 5,27 | 4,37 | 3,95 |
| **245** | 5,23 | 4,70 | 4,12 | **288** | 5,58 | 4,76 | 4,20 | **331** | 5,37 | 4,69 | 4,66 |
| **246** | 4,78 | 4,22 | 4,18 | **289** | 5,16 | 4,16 | 3,78 | **332** | 4,72 | 3,75 | 3,73 |
| **247** | 5,34 | 4,61 | 4,22 | **290** | 4,84 | 3,80 | 3,72 | **333** | 5,51 | 4,42 | 4,46 |
| **248** | 5,28 | 4,49 | 4,26 | **291** | 4,87 | 3,95 | 4,01 | **334** | 4,83 | 4,42 | 4,25 |
| **249** | 4,85 | 4,67 | 3,89 | **292** | 5,50 | 4,56 | 4,11 | **335** | 4,78 | 4,08 | 3,81 |
| **250** | 4,76 | 3,66 | 3,46 | **293** | 5,78 | 4,80 | 3,98 | **336** | 5,04 | 4,14 | 4,09 |
| **251** | 5,84 | 5,08 | 4,52 | **294** | 4,51 | 3,76 | 3,28 | **337** | 4,88 | 4,25 | 3,86 |
| **252** | 5,53 | 4,95 | 4,30 | **295** | 4,66 | 3,91 | 3,76 | **338** | 5,03 | 4,26 | 4,07 |
| **253** | 5,86 | 4,22 | 4,33 | **296** | 5,77 | 4,67 | 3,97 | **339** | 5,65 | 4,83 | 4,37 |
| **254** | 5,03 | 3,82 | 3,40 | **297** | 5,46 | 4,54 | 4,01 | **340** | 4,67 | 4,09 | 4,22 |
| **255** | 5,66 | 4,62 | 4,39 | **298** | 5,11 | 4,34 | 3,44 | **341** | 4,95 | 4,05 | 3,91 |
| **256** | 5,21 | 4,62 | 3,85 | **299** | 5,45 | 4,58 | 4,32 | **342** | 5,51 | 4,47 | 3,96 |
| **257** | 5,38 | 4,76 | 4,17 | **300** | 5,36 | 4,53 | 3,68 | **343** | 4,55 | 3,59 | 3,95 |
| **258** | 6,06 | 5,04 | 4,62 | **301** | 5,54 | 4,78 | 3,88 | **344** | 5,11 | 4,26 | 4,11 |
| **259** | 5,76 | 4,80 | 4,56 | **302** | 4,97 | 4,64 | 4,13 | **345** | 5,06 | 4,03 | 3,78 |
| **260** | 5,29 | 4,39 | 4,34 | **303** | 5,18 | 4,40 | 4,11 | **346** | 4,69 | 4,08 | 3,99 |
| **261** | 4,87 | 3,91 | 4,12 | **304** | 6,70 | 5,60 | 5,09 | **347** | 4,45 | 3,74 | 3,64 |
| **262** | 4,96 | 4,00 | 3,98 | **305** | 5,56 | 4,68 | 4,05 | **348** | 4,89 | 4,14 | 3,87 |
| **263** | 5,18 | 4,38 | 4,14 | **306** | 4,79 | 4,20 | 3,61 | **349** | 4,72 | 3,95 | 4,12 |
| **264** | 4,72 | 3,85 | 4,07 | **307** | 4,74 | 4,29 | 3,64 | **350** | 4,28 | 3,47 | 3,44 |
| **265** | 5,33 | 4,17 | 3,79 | **308** | 5,02 | 4,51 | 3,90 | **351** | 5,32 | 4,29 | 4,25 |
| **266** | 4,65 | 4,01 | 3,30 | **309** | 4,66 | 4,11 | 3,75 | **352** | 5,09 | 4,63 | 4,23 |
| **267** | 5,26 | 4,56 | 4,06 | **310** | 4,89 | 3,85 | 3,93 | **353** | 5,36 | 4,68 | 4,06 |
| **268** | 5,18 | 4,31 | 3,92 | **311** | 5,55 | 4,83 | 4,11 | **354** | 4,90 | 3,95 | 3,51 |
| **269** | 4,09 | 3,73 | 3,22 | **312** | 5,11 | 4,68 | 4,13 | **355** | 4,66 | 3,66 | 3,45 |
| **270** | 5,00 | 4,44 | 3,95 | **313** | 5,52 | 4,86 | 3,62 | **356** | 5,31 | 4,51 | 4,02 |
| **271** | 4,98 | 4,14 | 3,90 | **314** | 5,62 | 4,43 | 4,24 | **357** | 5,68 | 5,03 | 4,61 |
| **272** | 4,72 | 3,85 | 4,07 | **315** | 4,58 | 4,09 | 3,66 | **358** | 4,31 | 3,85 | 3,72 |
| **273** | 5,33 | 4,17 | 3,79 | **316** | 4,35 | 3,43 | 3,34 | **359** | 4,83 | 4,22 | 4,30 |
| **274** | 4,65 | 4,01 | 3,30 | **317** | 4,28 | 3,71 | 3,34 | **360** | 5,07 | 4,51 | 3,97 |
| **275** | 5,26 | 4,56 | 4,06 | **318** | 3,77 | 3,31 | 3,30 | **361** | 4,94 | 4,53 | 3,91 |
| **276** | 5,44 | 4,77 | 4,27 | **319** | 4,38 | 4,16 | 3,69 | **362** | 4,30 | 4,05 | 3,70 |
| **277** | 5,18 | 4,31 | 3,92 | **320** | 4,76 | 3,73 | 3,42 | **363** | 4,83 | 4,09 | 3,43 |
| **278** | 4,09 | 3,73 | 3,22 | **321** | 4,15 | 3,57 | 3,5 | **364** | 4,96 | 4,24 | 3,97 |
| **279** | 5,00 | 4,44 | 3,95 | **322** | 4,67 | 4,08 | 4,00 | **365** | 4,99 | 4,20 | 3,53 |
| **280** | 4,98 | 4,14 | 3,90 | **323** | 5,07 | 4,30 | 3,73 | **366** | 4,77 | 4,39 | 3,72 |
| **281** | 5,25 | 4,62 | 4,27 | **324** | 4,28 | 3,68 | 3,15 | **367** | 5,32 | 4,44 | 3,82 |
| **282** | 5,63 | 4,41 | 4,05 | **325** | 4,08 | 3,43 | 3,21 | **368** | 4,71 | 4,04 | 3,96 |
| **283** | 6,76 | 5,40 | 4,51 | **326** | 4,10 | 4,03 | 3,43 | **369** | 4,61 | 4,07 | 3,42 |
| **284** | 5,43 | 4,47 | 4,33 | **327** | 4,88 | 4,24 | 3,55 | **Average** | 5,04 | 4,29 | 3,93 |
|  |  |  |  |  |  |  |  | **SD(±)** | 0,49 | 0,41 | 0,36 |

**Continuation of Supplementary Table S3**

|  | Yiftah'el L715_F41 | | | | | | | | | | |
| --- | --- | --- | --- | --- | --- | --- | --- | --- | --- | --- | --- |
| **ID sample** | Length | Breadth | Thickness | **ID sample** | Length | Breadth | Thickness | **ID sample** | Length | Breadth | Thickness |
| **370** | 5,90 | 4,31 | 4,10 | **404** | 4,82 | 4,19 | 3,57 | **438** | 4,30 | 3,62 | 3,27 |
| **371** | 4,88 | 4,16 | 3,69 | **405** | 4,30 | 3,80 | 3,50 | **439** | 5,25 | 4,45 | 3,83 |
| **372** | 5,31 | 4,07 | 3,51 | **406** | 5,09 | 4,30 | 3,64 | **440** | 5,62 | 4,93 | 4,13 |
| **373** | 5,17 | 4,19 | 3,84 | **407** | 4,28 | 3,71 | 3,64 | **441** | 4,88 | 4,15 | 3,94 |
| **374** | 4,91 | 3,60 | 3,77 | **408** | 4,67 | 4,17 | 3,67 | **442** | 4,88 | 3,98 | 3,59 |
| **375** | 6,10 | 4,72 | 3,87 | **409** | 4,70 | 4,11 | 3,64 | **443** | 4,86 | 3,83 | 3,22 |
| **376** | 4,90 | 3,80 | 3,54 | **410** | 4,51 | 3,46 | 3,26 | **444** | 5,79 | 4,58 | 4,09 |
| **377** | 5,20 | 4,39 | 3,86 | **411** | 3,70 | 2,92 | 2,96 | **445** | 4,90 | 4,10 | 3,79 |
| **378** | 4,91 | 4,07 | 3,67 | **412** | 4,78 | 4,17 | 3,60 | **446** | 5,10 | 4,31 | 3,85 |
| **379** | 5,43 | 4,49 | 3,58 | **413** | 4,86 | 3,99 | 2,96 | **447** | 5,82 | 4,48 | 4,12 |
| **380** | 4,98 | 4,22 | 3,84 | **414** | 4,20 | 3,64 | 3,29 | **448** | 5,03 | 4,40 | 3,62 |
| **381** | 4,79 | 3,91 | 3,52 | **415** | 5,34 | 4,43 | 3,76 | **449** | 5,24 | 4,48 | 3,91 |
| **382** | 5,48 | 4,40 | 3,78 | **416** | 4,54 | 3,99 | 3,61 | **450** | 5,09 | 4,07 | 3,62 |
| **383** | 5,70 | 4,58 | 4,46 | **417** | 5,12 | 3,84 | 3,84 | **451** | 5,32 | 4,05 | 3,70 |
| **384** | 4,98 | 4,15 | 3,50 | **418** | 5,10 | 4,43 | 3,83 | **452** | 5,60 | 4,33 | 4,10 |
| **385** | 4,93 | 4,19 | 3,79 | **419** | 4,65 | 3,79 | 3,69 | **453** | 4,82 | 3,67 | 3,51 |
| **386** | 5,02 | 4,22 | 3,46 | **420** | 5,07 | 4,49 | 3,48 | **454** | 4,51 | 3,87 | 3,54 |
| **387** | 4,81 | 3,58 | 3,36 | **421** | 4,76 | 3,88 | 3,91 | **455** | 4,09 | 3,54 | 3,46 |
| **388** | 4,74 | 4,43 | 3,35 | **422** | 4,89 | 4,10 | 3,88 | **456** | 5,45 | 4,52 | 4,60 |
| **389** | 4,87 | 3,87 | 3,32 | **423** | 5,17 | 3,85 | 3,95 | **457** | 5,40 | 4,19 | 3,79 |
| **390** | 5,22 | 3,87 | 3,40 | **424** | 5,06 | 3,81 | 3,53 | **458** | 4,36 | 4,04 | 3,92 |
| **391** | 5,27 | 4,40 | 4,11 | **425** | 5,81 | 5,00 | 3,56 | **459** | 4,86 | 3,94 | 3,84 |
| **392** | 5,05 | 4,01 | 3,83 | **426** | 4,51 | 3,63 | 3,74 | **460** | 4,63 | 4,12 | 3,73 |
| **393** | 5,52 | 3,99 | 4,03 | **427** | 4,94 | 4,01 | 4,10 | **461** | 4,89 | 4,61 | 3,31 |
| **394** | 4,85 | 4,09 | 3,84 | **428** | 4,73 | 4,29 | 3,82 | **462** | 5,41 | 4,60 | 3,76 |
| **395** | 5,39 | 4,26 | 3,71 | **429** | 4,28 | 3,38 | 3,19 | **463** | 5,28 | 4,27 | 3,89 |
| **396** | 5,31 | 3,95 | 3,53 | **430** | 4,87 | 4,21 | 3,81 | **464** | 5,42 | 4,43 | \ |
| **397** | 5,18 | 4,06 | 3,84 | **431** | 3,94 | 3,95 | 3,45 | **465** | 5,42 | 4,45 | \ |
| **398** | 4,76 | 3,40 | 3,06 | **432** | 5,18 | 3,84 | 3,99 | **466** | 4,95 | 4,57 | \ |
| **399** | 5,31 | 4,44 | 3,42 | **433** | 4,75 | 4,15 | 3,68 | **467** | 5,21 | 4,95 | \ |
| **400** | 4,84 | 3,80 | 3,92 | **434** | 5,07 | 4,28 | 4,00 | **468** | 5,10 | 4,01 | \ |
| **401** | 4,72 | 4,17 | 3,34 | **435** | 5,12 | 4,35 | 3,89 | **469** | 4,65 | 3,71 | \ |
| **402** | 4,60 | 3,82 | 3,55 | **436** | 5,26 | 4,07 | 3,93 | **Average** | 4,99 | 4,11 | 3,69 |
| **403** | 4,19 | 3,74 | 3,32 | **437** | 5,32 | 4,64 | 4,03 | **SD(±)** | 0,43 | 0,36 | 0,29 |

**Supplementary Table S4| Results of the Z-test performed on the length of the archaeological *Vicia faba* L (*n* 469).** Differences between the mean values of the length of each context are considered statistically valid for P<0,001.

|  | AH L450_E14 | AH L398_D13 | AH L450_E14 | NZ L273_C11 | AH L450_E14 | YF 5073_G18 | AH L450_E14 | YF L715_F41 |
| --- | --- | --- | --- | --- | --- | --- | --- | --- |
| Mean (mm) | 6,13 | 5,90 | 6,13 | 5,22 | 6,13 | 5,04 | 6,13 | 4,99 |
| Known Variance | 0,21 | 0,3 | 0,21 | 0,28 | 0,21 | 0,23 | 0,21 | 0,18 |
| Observations | 128 | 77 | 128 | 36 | 128 | 128 | 128 | 100 |
| z | -3,088 |  | 9,284 |  | 18,502 |  | 19,406 |  |
| P(Z<=z) two-tail | 0,002 |  | 0 |  | 0 |  | 0 |  |
|  | AH L398_D13 | NZ L273_C11 | AH L398_D13 | L5073_G18 | AH L398_D13 | YF L715_F41 |  |  |
| Mean (mm) | 5,90 | 5,22 | 5,90 | 5,04 | 5,90 | 4,99 |  |  |
| Known Variance | 0,3 | 0,28 | 0,3 | 0,23 | 0,3 | 0,18 |  |  |
| Observations | 77 | 36 | 77 | 128 | 77 | 100 |  |  |
| z | 6,212 |  | 11,331 |  | 12,037 |  |  |  |
| P(Z<=z) two-tail | 0 |  | 0 |  | 0 |  |  |  |
|  | NZ L273_C11 | YF 5073_G18 | NZ L273_C11 | YF L715_F41 |  |  |  |  |
| Mean (mm) | 5,22 | 5,04 | 5,22 | 4,99 |  |  |  |  |
| Known Variance | 0,28 | 0,23 | 0,28 | 0,18 |  |  |  |  |
| Observations | 36 | 128 | 36 | 100 |  |  |  |  |
| z | 1,878 |  | 2,425 |  |  |  |  |  |
| P(Z<=z) two-tail | 0,06 |  | 0,015 |  |  |  |  |  |
|  | YF 5073_G18 | YF L715_F41 |  |  |  |  |  |  |
| Mean (mm) | 5,04 | 4,99 |  |  |  |  |  |  |
| Known Variance | 0,23 | 0,18 |  |  |  |  |  |  |
| Observations | 128 | 100 |  |  |  |  |  |  |
| z | 0,892 |  |  |  |  |  |  |  |
| P(Z<=z) two-tail | 0,372 |  |  |  |  |  |  |  |

**Supplementary Table S5| Radiocarbon dates and cultural chronology.**

| Site | Context | ID# | ^14^C  Uncal. BP | ^14^C  Cal. BP (1σ) | Modeled age  Cal. BP (1σ) | ^14^C  Cal. BCE (1σ) | Cultural Phase |
| --- | --- | --- | --- | --- | --- | --- | --- |
| Ahihud | L450_E14 | RTK-6866 | 9030±60 | 10250-10165 | 10235-10180 | 8345-7970 | EPPNB |
| Ahihud | L398_D13 | RTK-6875 | 8950±65 | 10205-9935 | 10215-10154 | 8290-7940 |
| Ahihud | L433_E14 | RTK-6868 | 8975±65 | 10230-9945 | 10205-10125 | 8280-8185 |
| Nahal Zippori 3 | L273_C11 | RTK-6864 | 8905±65 | 10175-9920 | 10160-9920 | 8260-7830 | MPPNB |
| Nahal Zippori 3 | L174_C11 | RTK-6865 | 8870±70 | 10170-9890 | 10160-9915 | 8215-7940 |
| Yiftah’el | L5073_G18 | RTK-6892 | 8895±65 | 10165-9915 | 10160-9920 | 8250-7795 |
| Yiftah’el | L715_F40 | RTK-6991 | 8815±40 | 10115-9735 | 10155-9890 | 8200-7740 |

**Supplementary Table S6| δ^13^C(‰) (and related Δ^13^C) values versus length (mm) of the 95 modern seeds of *Vicia faba* var. *minor***

|  | Ahihud L450_E14 | | | |  | Ahihud L398_D13 | | | |
| --- | --- | --- | --- | --- | --- | --- | --- | --- | --- |
| **ID sample** | δ^13^C | δ^13^C_air_ | Δ ^13^C | Length | **ID sample** | δ^13^C | δ^13^C_air_ | Δ ^13^C | Length |
| **1** | -25,50 | -6,70 | 19,29 | 6,35 | **20** | -24,90 | -6,70 | 18,66 | 5,50 |
| **2** | -23,90 | -6,70 | 17,62 | 6,45 | **21** | -24,80 | -6,70 | 18,56 | 6,70 |
| **3** | -24,90 | -6,70 | 18,66 | 6,00 | **22** | -22,90 | -6,70 | 16,58 | 6,20 |
| **4** | -24,50 | -6,70 | 18,25 | 5,70 | **23** | -24,40 | -6,70 | 18,14 | 5,60 |
| **5** | -25,80 | -6,70 | 19,61 | 6,10 | **24** | -25,00 | -6,70 | 18,77 | 6,30 |
| **6** | -25,30 | -6,70 | 19,08 | 6,45 | **25** | -22,40 | -6,70 | 16,06 | 6,20 |
| **7** | -25,20 | -6,70 | 18,98 | 6,25 | **26** | -25,00 | -6,70 | 18,77 | 6,30 |
| **8** | -24,90 | -6,70 | 18,66 | 6,40 | **27** | -24,40 | -6,70 | 18,14 | 6,60 |
| **9** | -24,80 | -6,70 | 18,56 | 5,85 | **28** | -22,90 | -6,70 | 16,58 | 6,00 |
| **10** | -23,80 | -6,70 | 17,52 | 6,80 | **29** | -24,10 | -6,70 | 17,83 | 5,60 |
| **11** | -24,10 | -6,70 | 17,83 | 6,90 | **30** | -24,00 | -6,70 | 17,73 | 5,85 |
| **12** | -24,00 | -6,70 | 17,73 | 5,85 | **31** | -22,90 | -6,70 | 16,58 | 6,05 |
| **13** | -23,80 | -6,70 | 17,52 | 5,90 | **32** | -25,50 | -6,70 | 19,29 | 5,45 |
| **14** | -24,70 | -6,70 | 18,46 | 5,30 | **33** | -24,60 | -6,70 | 18,35 | 5,65 |
| **15** | -23,80 | -6,70 | 17,52 | 4,90 | **34** | -24,30 | -6,70 | 18,04 | 5,65 |
| **16** | -23,70 | -6,70 | 17,41 | 6,00 | **35** | -25,50 | -6,70 | 19,29 | 5,25 |
| **17** | -25,10 | -6,70 | 18,87 | 6,05 | **36** | -25,10 | -6,70 | 18,87 | 5,10 |
| **18** | -24,90 | -6,70 | 18,66 | 6,70 | **37** | -24,40 | -6,70 | 18,14 | 6,10 |
| **19** | -24,60 | -6,70 | 18,35 | 6,55 | **38** | -25,40 | -6,70 | 19,19 | 5,60 |
| **Average** | -24,59 | -6,70 | 18,35 | 6,13 | **Average** | -24,34 | -6,70 | 18,08 | 5,88 |
| **SD(±)** | 0,65 | 0 | 0,67 | 0,50 | **SD(±)** | 0,94 | 0 | 0,98 | 0,44 |

| Nahal Zippori 3 L273_C11 | | | | |
| --- | --- | --- | --- | --- |
| **ID sample** | δ^13^C | δ^13^C_air_ | Δ ^13^C | Length |
| **39** | -25,10 | -6,69 | 18,88 | 5,80 |
| **40** | -23,50 | -6,69 | 17,21 | 5,30 |
| **41** | -23,70 | -6,69 | 17,42 | 5,70 |
| **42** | -25,00 | -6,69 | 18,78 | 5,25 |
| **43** | -25,10 | -6,69 | 18,88 | 5,35 |
| **44** | -25,50 | -6,69 | 19,30 | 5,35 |
| **45** | -23,50 | -6,69 | 17,21 | 5,35 |
| **46** | -23,30 | -6,69 | 17,11 | 4,80 |
| **47** | -25,10 | -6,69 | 18,88 | 4,95 |
| **48** | -23,70 | -6,69 | 17,42 | 5,50 |
| **49** | -23,10 | -6,69 | 16,80 | 5,91 |
| **50** | -23,30 | -6,69 | 17,01 | 5,31 |
| **51** | -24,40 | -6,69 | 18,15 | 5,30 |
| **52** | -24,00 | -6,69 | 17,74 | 5,00 |
| **53** | -23,90 | -6,69 | 17,63 | 4,85 |
| **54** | -24,60 | -6,69 | 18,36 | 5,60 |
| **55** | -24,00 | -6,69 | 17,74 | 6,20 |
| **56** | -23,90 | -6,69 | 17,63 | 5,45 |
| **57** | -23,70 | -6,69 | 17,42 | 4,99 |
| **Average** | -24,13 | -6,69 | 17,87 | 5,33 |
| **SD(±)** | 0,73 | 0 | 0,76 | 0,37 |

**Continuation of Supplementary Table S6**

|  | Yiftah'el L5073_G18 | | | |  | Yiftah'el L715_F41 | | | |
| --- | --- | --- | --- | --- | --- | --- | --- | --- | --- |
| **ID sample** | δ^13^C | δ^13^C_air_ | Δ ^13^C | Length | **ID sample** | δ^13^C | δ^13^C_air_ | Δ ^13^C | Length |
| **58** | -25,00 | -6,68 | 18,80 | 5,90 | **77** | -23,60 | -6,67 | 17,33 | 5,35 |
| **59** | -22,90 | -6,68 | 16,61 | 4,90 | **78** | -22,30 | -6,67 | 15,98 | 5,15 |
| **60** | -22,10 | -6,68 | 15,78 | 5,30 | **79** | -23,50 | -6,67 | 17,22 | 5,45 |
| **61** | -22,50 | -6,68 | 16,19 | 5,15 | **80** | -22,70 | -6,67 | 16,39 | 5,15 |
| **62** | -24,30 | -6,68 | 18,07 | 4,90 | **81** | -22,60 | -6,67 | 16,29 | 4,90 |
| **63** | -22,70 | -6,68 | 16,40 | 5,25 | **82** | -22,60 | -6,67 | 16,29 | 5,45 |
| **64** | -22,30 | -6,68 | 15,99 | 4,80 | **83** | -23,60 | -6,67 | 17,33 | 6,15 |
| **65** | -21,90 | -6,68 | 15,57 | 4,80 | **84** | -23,20 | -6,67 | 16,91 | 4,90 |
| **66** | -21,90 | -6,68 | 15,57 | 5,00 | **85** | -24,50 | -6,67 | 18,27 | 5,25 |
| **67** | -21,60 | -6,68 | 15,26 | 5,40 | **86** | -23,50 | -6,67 | 17,22 | 4,95 |
| **68** | -22,90 | -6,68 | 16,60 | 5,15 | **87** | -23,30 | -6,67 | 17,02 | 5,10 |
| **69** | -24,40 | -6,68 | 18,16 | 4,85 | **88** | -23,40 | -6,67 | 17,13 | 4,75 |
| **70** | -24,10 | -6,68 | 17,85 | 5,50 | **89** | -23,50 | -6,67 | 17,24 | 4,80 |
| **71** | -21,50 | -6,68 | 15,15 | 4,80 | **90** | -23,70 | -6,67 | 17,44 | 4,20 |
| **72** | -23,10 | -6,68 | 16,81 | 4,90 | **91** | -22,00 | -6,67 | 15,67 | 4,90 |
| **73** | -23,50 | -6,68 | 17,22 | 4,35 | **92** | -24,60 | -6,67 | 18,38 | 5,05 |
| **74** | -22,30 | -6,68 | 15,98 | 4,30 | **93** | -24,30 | -6,67 | 18,07 | 5,80 |
| **75** | -22,90 | -6,68 | 16,60 | 4,45 | **94** | -24,50 | -6,67 | 18,28 | 4,50 |
| **76** | -22,30 | -6,68 | 15,98 | 4,30 | **95** | -24,50 | -6,67 | 18,28 | 4,95 |
| **Average** | -22,85 | -6,68 | 16,56 | 4,95 | **Average** | -23,47 | -6,67 | 17,20 | 5,09 |
| **SD(±)** | 1,00 | 0 | 1,04 | 0,42 | **SD(±)** | 0,78 | 0 | 0,82 | 0,44 |
|  |  | | | |  |  | | | |

**Supplementary Table S7| Results of the Z-test performed on the Δ^13^C (‰) of the archaeological *Vicia faba* L. (*n* 95).** Differences between the mean values of the Δ^13^C of each context are considered statistically valid for P<0,001.

|  | AH L450_E14 | AH L398_D13 | AH L450_E14 | NZ L273_C11 | AH L450_E14 | YF 5073_G18 | AH L450_E14 | YF L715_F41 |
| --- | --- | --- | --- | --- | --- | --- | --- | --- |
| Mean | 18,35 | 18,08 | 18,35 | 17,87 | 18,35 | 16,56 | 18,35 | 17,20 |
| Known Variance | 0,45 | 0,96 | 0,45 | 0,57 | 0,45 | 1,08 | 0,45 | 0,67 |
| Observations | 19 | 19 | 19 | 19 | 19 | 19 | 19 | 19 |
| z | 0,966 |  | 2,042 |  | 6,304 |  | 4,734 |  |
| P(Z<=z) two-tail | 0,334 |  | 0,041 |  | 0 |  | 0 |  |
|  | AH L398_D13 | NZ L273_C11 | AH L398_D13 | L5073_G18 | AH L398_D13 | YF L715_F41 |  |  |
| Mean | 18,08 | 17,87 | 18,08 | 16,56 | 18,08 | 17,20 |  |  |
| Known Variance | 0,96 | 0,57 | 0,96 | 1,08 | 0,96 | 0,67 |  |  |
| Observations | 19 | 19 | 19 | 19 | 19 | 19 |  |  |
| z | 0,740 |  | 4,656 |  | 3,026 |  |  |  |
| P(Z<=z) two-tail | 0,459 |  | 0 |  | 0,002 |  |  |  |
|  | NZ L273_C11 | YF 5073_G18 | NZ L273_C11 | YF L715_F41 |  |  |  |  |
| Mean | 17,87 | 16,56 | 17,87 | 17,20 |  |  |  |  |
| Known Variance | 0,57 | 1,08 | 0,57 | 0,67 |  |  |  |  |
| Observations | 19 | 19 | 19 | 19 |  |  |  |  |
| z | 4,465 |  | 2,647 |  |  |  |  |  |
| P(Z<=z) two-tail | 0 |  | 0,008 |  |  |  |  |  |
|  | YF 5073_G18 | YF L715_F41 |  |  |  |  |  |  |
| Mean | 16,56 | 17,20 |  |  |  |  |  |  |
| Known Variance | 1,08 | 0,67 |  |  |  |  |  |  |
| Observations | 19 | 19 |  |  |  |  |  |  |
| z | -2,107 |  |  |  |  |  |  |  |
| P(Z<=z) two-tail | 0,035 |  |  |  |  |  |  |  |

**Supplementary S8. The contexts of findings**

*Vicia faba* L. was collected from secure contexts in three Pre- Pottery Neolithic B (PPNB) sites, Ahihud, Yiftah’el and Nahal Zippori 3.

**Ahihud**. The site was discovered and excavated in 2013 by two of the authors (Y.P. and J.V.) on behalf of the Israel Antiquities Authority^7^. The site is situated on the northern slope of Ahihud Hill at the eastern edge of the ʿAkko (Acre) valley, northern Israel. The archaeological investigations at the site exposed remains of two settlements partially superimposed: Late Pottery Neolithic (Wadi Raba culture) and Pre-Pottery Neolithic B from where the legumes reported in this paper were recovered. The PPNB layer is composed of beaten earth floors that were placed on top of levelled cobble fills. These surfaces were constructed on the natural bedrock that is characterized by numerous fissures and depressions and shows evidence for quarrying.

The lithics from the PPNB contexts include a typical PPNB tool kit with formal types such as arrowheads and sickle blades that were fabricated on bidirectional (naviform) blades. Observations on the lithic components suggest that the major occupational phase is the Early PPNB. This relies mainly on the dominance of Helwan and Jericho types^7^ among the arrowheads and by the presence of *tranchet* axes and transversal spalls^56-58^. In addition to the flint, ca. 100 obsidian artifacts were recovered. Notably some are Helwan points, also found at other Early PPNB sites within the southern Levant, such as Motza; Nahal Lavan 109^59-62^. PXRF analysis shows the obsidian source was Bingol, Anatolia (E. Rice personal communication).

The pulses that presented in this paper were almost entirely retrieved from two silos that were dug in the ground (**Fig. S1**). Silo L 450, in square E14, was sealed by an Early PPNB floor that totally covered a massive wall whose lower coarse partially penetrated the seed rich layer. The second silo, L 398, in square D13, was associated with another living floor. More specifically, 50 liters of sediments were collected from L 450, and 30 liters from L 398. Seeds were separated from the rest of the sediment using sieves of 1 mm mesh, on the whole, 2043 seeds + 4162 fragments were collected. Notably, additional 200 liters of sediment, coming from the two investigated contexts, have not been sieved yet. Therefore it is likely that the number of fully preserved seeds should increase.
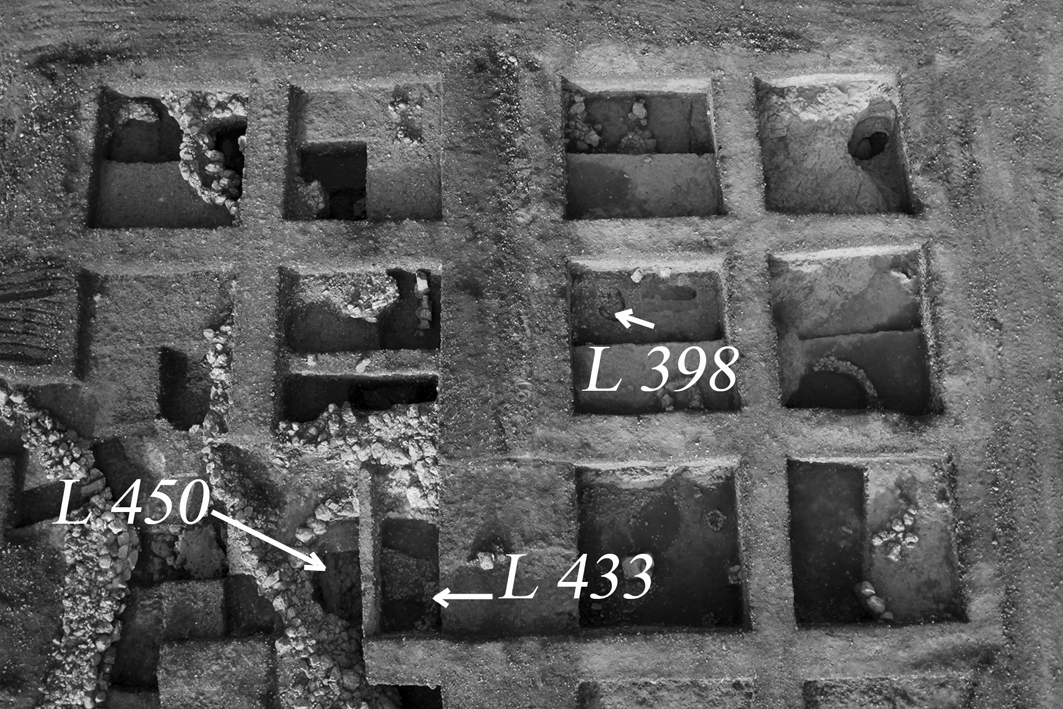


Figure S1. Location of the storage pits L 450 and L 398 and L 433 at Ahihud.

**Yiftah’el**. It is a late prehistoric site with an important PPNB occupation extending over ca. 4 hectares^8^. It was excavated by several expeditions who exposed numerous PPNB contexts containing buildings and domestic installations in different areas. Most of the buildings could be considered as domestic (e.g. Areas C, D, E and I), but some seem to have been used for cultic purposes (Area G). Building techniques include construction of mud-brick and field stone walls and plastered floors. Finds include mainly lithic artifacts, of which many were produced by bidirectional blade technology. The formal tools consist of sickle blades and arrowheads (mainly Jericho and Byblos points) and bifacial tools characteristics of the Middle and probably of the Mid-Late PPNB^8,60^. Other stone tools include small polished axes made of greenstones^60^. The archaeobotanical remains at the site show an outstanding preservation with charred legume seeds found in several silos and find spot concentrations in Areas C, E and I. The specimens presented here were from L 715, a pit with seeds in Area C published before^62,63^, and from an unpublished debris layer in Area I (L 5073) (**Fig. S2**), handpicked by one of the authors (E.B.). On the whole, for this study 796 seeds + 273 fragments were collected.

**
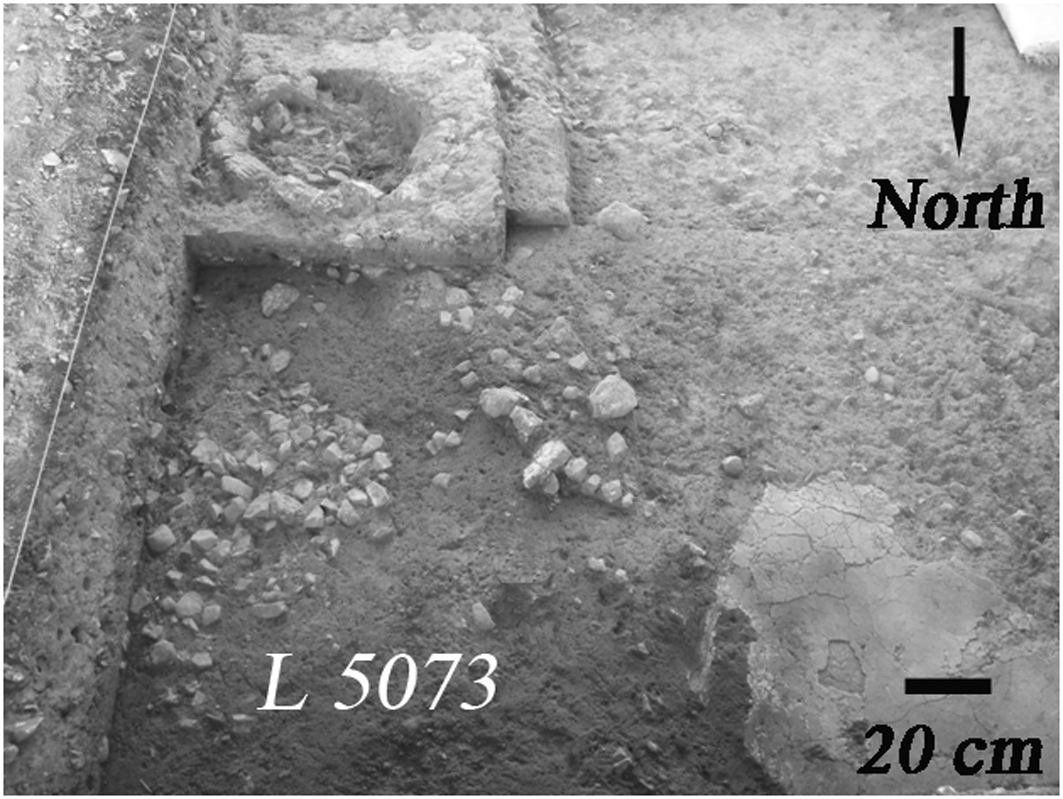
**

Figure S2. Location of the L 5073 in Area I at Yiftah’el.

**Nahal Zippori 3.** The PPNB settlement of Nahal Zippori 3 shows similar characteristics to its neighboring site Yiftahel^9,19^. It contains remains of several domestic buildings and a few constructed installations. Like in Yiftahel the structures were built from mud-brick walls and lime-plastered floors. The portable finds consist of lithics, ground stone tools, and bone tool assemblages. The lithic assemblage is characterized by three technologies: bidirectional blades, bifacial tools and *ad hoc*. Notable is the presence of Jericho and Byblos points, inversely retouched sickle blades and polished axes which suggest that the major occupational phase was during the Middle PPNB^9,64^. All charred seeds, identified as legumes, were found inside a building (**Fig. S3**). The seeds used for the present work were collected from a depression on the plastered floor L 273 in square C11, Layer 5, but several others were found within the same building on plaster floor L 268 and pit L 271^19^. All specimens were handpicked in the field for a total number of 89 seeds + 43 fragments .
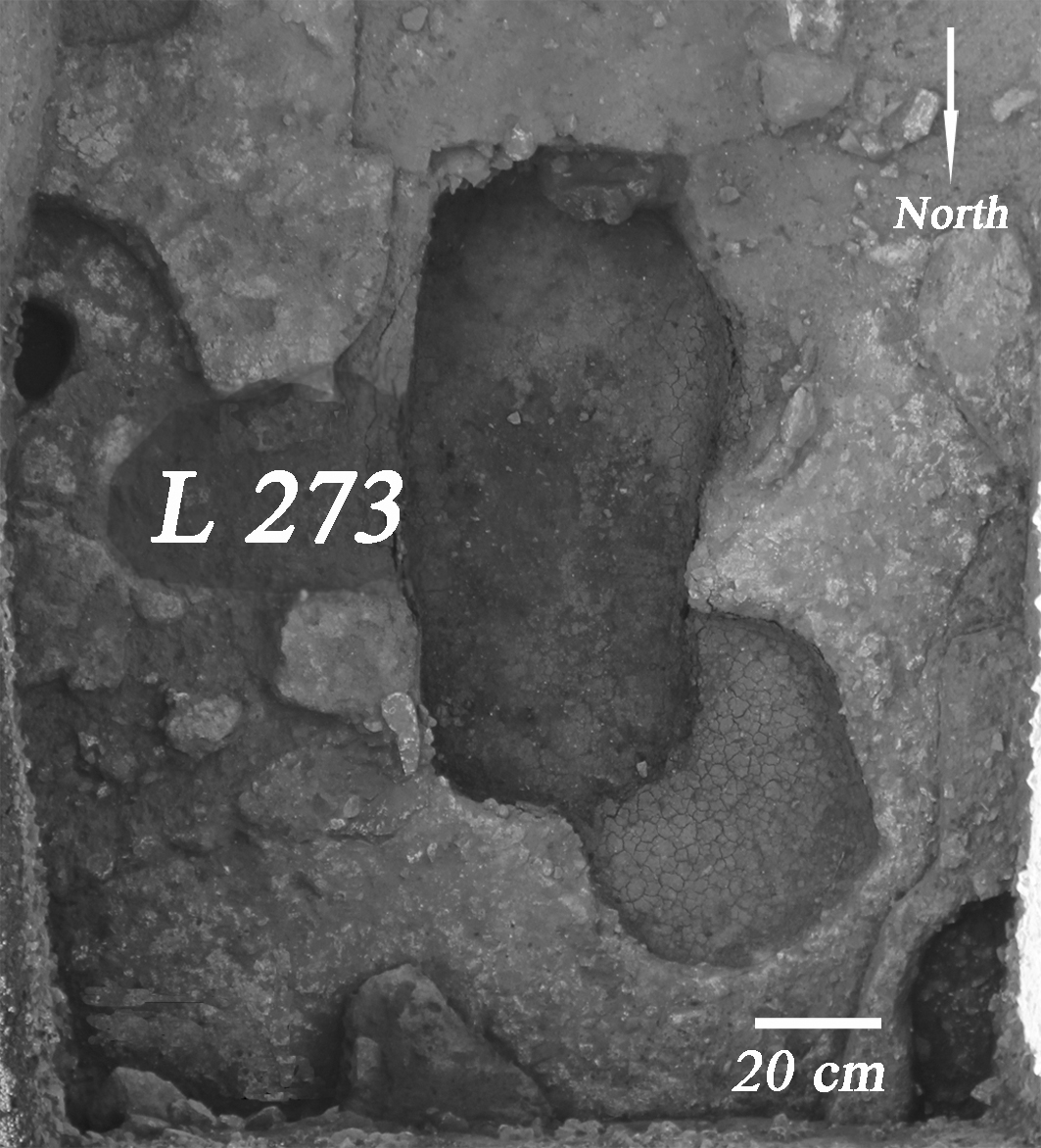


Figure S3. The plaster floor L 273 in the site of Nahal Zippori 3 (Photo by Assaf Peretz, courtesy of Israel Antiquities Authority).

**References**

56. Gopher, A. *Arrowheads of the Neolithic Levant*. ASOR Dissertation Series 10. (Eisenbrauns, Winona Lake, 1994).

57. Kuijt, I., Goring-Morris, N., Foraging, farming and social complexity in the Pre-Pottery Neolithic of the southern-central Levant: a review and synthesis. *J. of World Prehist*. **16**, 361-440 (2002).

58. Barkai, R. *Flint and Stone Axes as Cultural Markers: Socio-Economic Changes as Reflected in Holocene Flint Tool Industries of the Southern Levant*. SENEPSE 11. (Ex Orient, Berlin, 2005).

59. Burian F *et al.* In *Materialien zur Vor- und Fruhgeschichte von Hessen 8*, 95–120 (Festchrift für Gunnter Smolla, Wiesbaden, 1999).

60. Khalaily, H. *et al* Excavation at Motza in the Judean Hills and the Early Pre-Pottery Neolithic B in the southern Levant. *Paléorient* **33**, 5–37 (2007).

61. Khalaily, H. *et al.* In *Stone Tools in Transition: From Hunter-Gatherers to Farming Societies in the Near East*, eds. Borrell Tena F et al*.* (Publicacions de la Universitat Autònoma de Barcelona, 2013).

62. Garfinkel, Y. In *The Pre-Pottery Neolithic B village of Yiftah’el,* 3-11 ed. Garfinkel Y et al. (Berlin Ex Oriente, 2012)

63. Garfinkel, Y. *et al.* Lentil in the pre-pottery Neolithic B Yiftah’el: additional evidence of its early domestication. *Isr. J. of Bot.* **37**, 49-51 (1988).

64. Barzilai, O. *Social Complexity in the Southern Levantine PPNB as Reflected through Lithic Studies: the Bidirectional Blade Industries* (BAR IS 2180, Oxford, 2010).
